# PD‐1^+^CD8^+^ T Cell‐Mediated Hepatocyte Pyroptosis Promotes Progression of Murine Autoimmune Liver Disease

**DOI:** 10.1002/advs.202407284

**Published:** 2024-11-04

**Authors:** Jie Long, Si‐Yu Yang, Zhen‐Hua Bian, Hao‐Xian Zhu, Min Ma, Xiao‐Qing Wang, Liang Li, Weici Zhang, Ying Han, M. Eric Gershwin, Zhe‐Xiong Lian, Zhi‐Bin Zhao

**Affiliations:** ^1^ Medical Research Institute, Guangdong Provincial People's Hospital (Guangdong Academy of Medical Sciences) Southern Medical University Guangzhou 510080 China; ^2^ Guangdong Cardiovascular Institute, Guangdong Provincial People's Hospital (Guangdong Academy of Medical Sciences) Southern Medical University Guangzhou 510080 China; ^3^ School of Biomedical Sciences and Engineering South China University of Technology Guangzhou International Campus Guangzhou 511442 China; ^4^ School of Medicine South China University of Technology Guangzhou 510006 China; ^5^ Guangdong Provincial People's Hospital (Guangdong Academy of Medical Sciences) Southern Medical University Guangzhou 510080 China; ^6^ Division of Rheumatology, Allergy and Clinical Immunology University of California Davis Davis CA 95616 USA; ^7^ State Key Laboratory of Cancer Biology, National Clinical Research Center for Digestive Diseases and Xijing Hospital of Digestive Diseases Air Force Military Medical University Xi'an 710000 China

**Keywords:** aire, autoimmune liver disease, dnTGFβRII, PD‐1^+^CD8^+^ T cells, pyroptosis

## Abstract

The specific mechanisms underlying effector pathways in autoimmune liver disease remain enigmatic and therefore constructing appropriate murine models to investigate disease pathogenesis becomes critical. A spontaneous severe murine model of autoimmune liver disease has been previously established in dnTGFβRII Aire^−/−^ mice, exhibiting disease phenotypes that resemble both human primary biliary cholangitis (PBC) and autoimmune hepatitis (AIH). The data suggests that auto‐reactive liver‐specific CD8^+^ T cells are the primary pathogenic cells in liver injury. In this study, these data are advanced through the use of both single‐cell sequencing and extensive in vitro analysis. The results identify a specific expanded pathogenic subset of PD‐1^+^CD8^+^ T cells in the liver, exhibiting strong functional activity and cytotoxicity against target cells. Depletion of PD‐1^+^CD8^+^ T cells using CAR‐T cells effectively alleviates the disease. GSDMD‐mediated pyroptosis is found to be aberrantly activated in the livers of model mice, and treatment with a GSDMD‐specific inhibitor significantly inhibits disease progression. In vitro experiments reveal that PD‐1^+^CD8^+^ T cells can induce the pyroptosis of hepatocytes through elevated production of granzyme B and perforin‐1. These results provide a novel explanation for the cytotoxic activity of pathogenic liver PD‐1^+^CD8^+^ T cells in autoimmune liver diseases and offer potential therapeutic targets.

## Graphical abstract

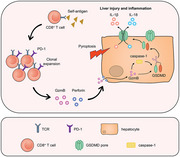

## 1 Introduction

Elevated expression of PD‐1 on the surface of CD8^+^ T cells is a characteristic of exhausted T cells. These T cells also express high levels of immune checkpoint molecules, including TIM3 and LAG3.^[^ ^1^ ^]^ Such PD‐1^+^CD8^+^ T cells exhibit weakened effector function and undergo apoptosis.^[^ ^2^ ^]^ In tumors, the increase of PD‐1^+^CD8^+^ T cells is closely associated with tumor immune evasion. Anti‐PD‐1 therapy has been supported to promote expansion of CD8^+^ T cells and production of cytokines.^[^ ^3^ ^]^ However, recent studies suggest that this view is not entirely accurate. A significant number of PD‐1^+^CD8^+^ T cells with an effector memory phenotype and function are present in the peripheral blood of healthy adults, which is markedly different from the traditional concept of HIV‐induced exhausted T cells.^[^ ^4^ ^]^ There is limited research on PD‐1^+^CD8^+^ T cells in autoimmune liver diseases, but recent studies have found a significant expansion of PD‐1^+^CD8^+^ T cells in the livers of patients with autoimmune hepatitis (AIH). Moreover, these PD‐1^+^CD8^+^ T cells demonstrate a positive correlation with serum alanine transaminase (ALT) and aspartate transaminase (AST) levels.^[^ ^5^ ^]^

Pyroptosis is a newly identified form of programmed cell death primarily mediated by caspase‐1/3, involving the release of proinflammatory factors and a cascade amplification of inflammatory responses.^[^ ^6^ ^]^ Activation of inflammatory caspases can further cleave intracellular gasdermins proteins, releasing N‐terminal pore‐forming regions. Subsequently, these regions bind to phospholipids on the cell membrane, oligomerize to form membrane pores, and facilitate the release of proinflammatory cytokines such as interleukin‐1β (IL‐1β).^[^ ^7^ ^]^

dnTGFβRII Aire^−/−^ mice spontaneously develop severe autoimmune liver disease, exhibiting symptoms of both PBC and AIH, with CD8^+^ T cells as the pathogenic effector cells.^[^ ^8^ ^]^ Herein, we report that among CD8^+^ T cells, the specifically expanded PD‐1^+^CD8^+^ T cells in liver is a critical pathogenic subset. Despite expressing high levels of immune checkpoint molecules, these PD‐1^+^CD8^+^ T cells exhibit heightened cytokine production and functional activity, displaying potent cytotoxic capabilities against target cells. Furthermore, PD‐1^+^CD8^+^ liver T cells induce GSDMD‐mediated pyroptosis in hepatocytes by producing high levels of granzyme B and perforin‐1. Our results provide a novel insight for potential therapeutic targets.

## 2 Results

### 2.1 Single Cell RNA Sequencing Underscore the Expansion of Activated CD8^+^ T Cell Clusters in dnTGFβRII Aire^−/−^ Mice

**Figure 1:**
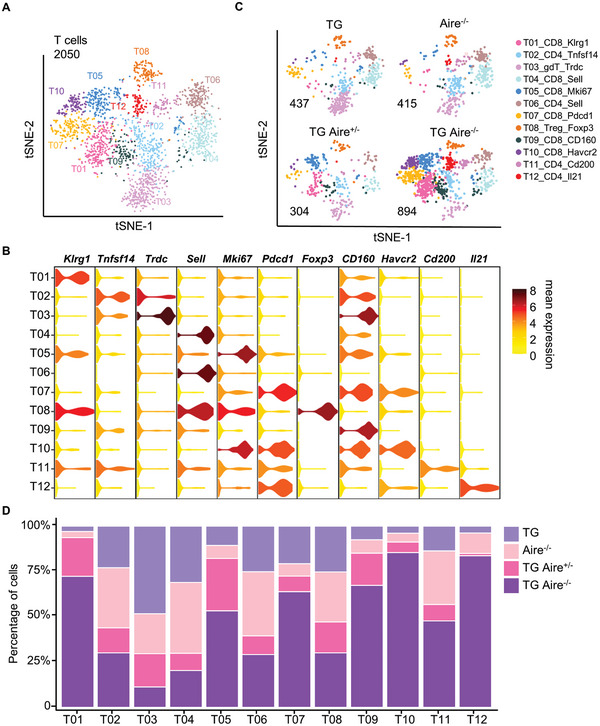
Single cell RNA sequence data underscore the expansion of activated CD8^+^ T cell clusters in TG Aire^−/−^ mice. A) Merged t‐SNE plot of single cell RNA sequence data of hepatic *Cd3d* ^+^ *Klra1* ^−^ T cells from TG, Aire^−/−^, TG Aire^+/−^ and TG Aire^−/−^ mice. The number in the top left corner indicates the number of cells. B) Combined violin plots of specific marker genes across 12 T cell clusters. C) Split t‐SNE plots of (A). The number in the bottom left corner indicates the number of cells. D) Relative frequency distribution of each T cell cluster in the livers from TG, Aire^−/−^, TG Aire^+/−^ and TG Aire^−/−^ mice. dnTGFβRII is abbreviated as TG.

To investigate whether there are pathogenic CD8^+^ T cell subsets in dnTGFβRII Aire^−/−^ mice, we performed a targeted single cell RNA‐seq (397 immune related target genes) of hepatic lymphocytes isolated from 2–4 week‐old dnTGFβRII Aire^−/−^, dnTGFβRII Aire^+/−^, dnTGFβRII and Aire^−/−^ mice. After quality control and filtering of the sequencing data, we obtained a data matrix consisting of 385 genes and 12923 cells. Subsequently, we selected 2050 *Cd3d* ^+^ *Klra1* ^−^ T cells for further analysis (Figure S1A‐D, Supporting Information). The t‐SNE plot of total T cells displayed 12 clusters which were composed of 6 clusters of CD8^+^ T cell, 5 clusters of CD4^+^ T cell and 1 cluster of γδT cell based on the expression levels of target genes (**Figure** **1**A). According to the marker genes of each cluster, we defined the CD8^+^ T clusters as follows: a) T01_CD8_Klrg1 that highly expressed several functional molecules *Klrg1*, *Gzma*, and *Itgax*, b) T04_CD8_Sell that expressed high levels of many naïve/central memory marker genes, like *Sell*, *Tcf7*, *Ccr7*, and *Il7r*, c) T05_CD8_Mki67 that expressed many cell cycle related gene, including *Mki67*, *Top2a*, *Aurkb* and *Tyms*, d) Two *Pdcd1* positive clusters, including T07_CD8_Pdcd1 and T10_CD8_Havcr2 that expressed high levels of many immune checkpoints, such as *Pdcd1*, *Havcr2*, *Lag3*, *Tigit*, and *Tnfrsf9*, and e) T09_CD8_CD160 that highly expressed *Cd160*, *Icam1*, and *Eomes* (Figure 1B). The split t‐SNE plots of samples demonstrated that the proportions of CD8^+^ T clusters including T01_CD8_Klrg1, T05_CD8_Mki67, T07_CD8_Pdcd1, T09_CD8_CD160, and T10_CD8_Havcr2, were significantly increased in the livers of dnTGFβRII Aire^−/‐^ mice as compared with dnTGFβRII Aire^+/−^, dnTGFβRII and Aire^−/−^ mice, except for T04_CD8_Sell that showed a lower proportion than dnTGFβRII and Aire^−/−^ mice (Figure 1C,D). There was also a small CD4^+^ T cluster that showed enrichment in the dnTGFβRII Aire^−/‐^ mice. This CD4^+^ T cluster expressed high levels of several important functional molecules, such as *Il10*, *Il21*, *Ifng*, and *Icos*. All other CD4^+^ T clusters including Treg cells had no significant differences between the livers of the 4 groups of mice (Figure 1B‐D).

### 2.2 Single‐Cell TCR Sequencing Reveals Clonal Expansion of Hepatic *Pdcd1* ^+^CD8^+^ T Cells in dnTGFβRII Aire^−/−^ Mice

**Figure 2:**
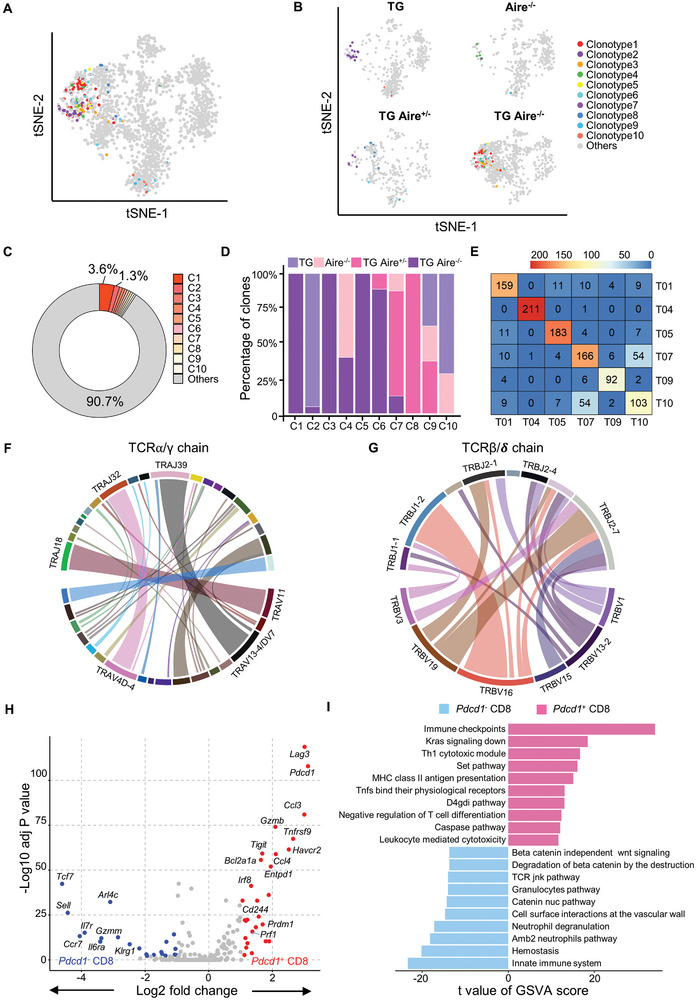
Single T cell receptor repertoire analysis and specific clonal expansion of *Pdcd1* ^+^CD8^+^ T clusters. A) Top 10 TCR clonotypes distributed on merged t‐SNE plot of hepatic T cells in TG, Aire^−/−^, TG Aire^+/−^ and TG Aire^−/−^ mice. B) Split t‐SNE plots from (A). C) Circular ring plot displays the frequency of top 10 TCR clones in total T cell clones. D) The relative frequency of top 10 TCR clones. E) Heatmap of shared TCR clonotypes between CD8^+^ T cells clusters; shared cell numbers are marked in the grids. The combinations of V and J genes in F) TCRα/γ chains and G) TCRβ/𝜹 chains. H) Volcano plot comparing differentially expressed genes between *Pdcd1* positive and negative CD8^+^ T cells. I) GSVA analysis displays the top 20 differentially enriched pathways between *Pdcd1* positive and negative CD8^+^ T cells. A non‐parameteric Wilcoxon rank sum test was performed to assess the statistical significance in (H).

Next, we performed a single cell TCR‐seq of sorted lymphocytes from the livers of dnTGFβRII, Aire^−/−^, dnTGFβRII Aire^+/−^, and dnTGFβRII Aire^−/−^ mice. A comprehensive analysis of the single cell RNA‐seq and TCR‐seq data demonstrated that most of the top 10 TCR clones were enriched in two *Pdcd1* ^+^ *Cd8a* ^+^ T clusters (T07_CD8_Pdcd1 and T10_CD8_Havcr2), which were massively expanded in dnTGFβRII Aire^−/‐^ mice while only a few cells with these clones existed in T03_gdT_Trdc and other CD8^+^ T cell clusters (**Figure** **2**A). The CDR3 amino acid sequence combination of the most expanded TCR clone is AMNNNAGAKLT || ASSTGGSDYT (TCRα||ΤCRβ), noted only in the livers of dnTGFβRII Aire^−/‐^ mice and accounts for up to 3.6% of all TCR clones; the total percentage of top 10 TCR clones was 9.3%. Furthermore, the C3 (AAEHYGSSGNKLI || ASSTGGEQY) and C5 (AASAQWRQQLQTD || ASSTGGSDYT) clonotypes were also only noted in livers from the dnTGFβRII Aire^−/−^ mice (Figure 2B‐D; Table S1, Supporting Information). We also found many TCR clones in T07_CD8_Pdcd1 and T10_CD8_Havcr2 overlapped (Figure 2E). The most frequently used V‐J gene recombination of TCRα/γ and TCRβ/δ were TRAV13‐4/DV7 || TRAJ39 (Figure 2F) and TRBV16 || TRBJ1‐2 (Figure 2G) respectively, which corresponds to the C1 clonotype (data not shown). The differentially expressed genes analysis indicated that *Pdcd1* ^+^ *Cd8a* ^+^ T cells expressed higher levels of immune checkpoint molecules including *Lag3*, *Pdcd1*, *Havcr2*, *Tnfrsf9*, and *Tigit*, as well as higher expression of *Prf1*, *Ccl3*, *Ccl4*, and *Gzmb* (Figure 2H). GSVA analysis revealed that several important pathways were upregulated in *Pdcd1* ^+^ *Cd8a* ^+^ T cells compared to *Pdcd1* ^−^ *Cd8a* ^+^ T cells, including the Th1 cytotoxic module and leukocyte mediated cytotoxicity pathways (Figure 2I). In addition, the analysis of differentially expressed genes between two distinct *Pdcd1* ^+^ *Cd8a* ^+^ T cell clusters revealed the T10_CD8_Havcr2 expressed significantly higher levels of many proliferation genes compared to T07_CD8_Pdcd1, including *Top2a*, *Mki67*, and *Tyms*, as well as higher levels of *Havcr2* (Figure S2A, Supporting Information). GSVA analysis suggested that many proliferation related pathways, including G2M checkpoint, Fischer G2M cell cycle and mitotic metaphase and anaphase pathways were upregulated in T10_CD8_Havcr2 compared to T07_CD8_Pdcd1 (Figure S2B, Supporting Information). Furthermore, these two *Pdcd1* ^+^ *Cd8a* ^+^ T cell clusters both expressed high levels of *Gzmb*, *Prf1*, *Ifng*, while *Tcf7* was nearly undetectable (Figure S2C, Supporting Information). These results indicate that *Pdcd1* ^+^ *Cd8a* ^+^ T cells are the predominant clonally expanded subsets in the livers of dnTGFβRII Aire^−/−^ mice, suggesting that these populations of cells may represent the critical pathogenic CD8^+^ T cell subsets.

### 2.3 Specifically Expanded PD‐1^+^CD8^+^ T Cells in the Livers of dnTGFβRII Aire^−/−^ Mice

**Figure 3:**
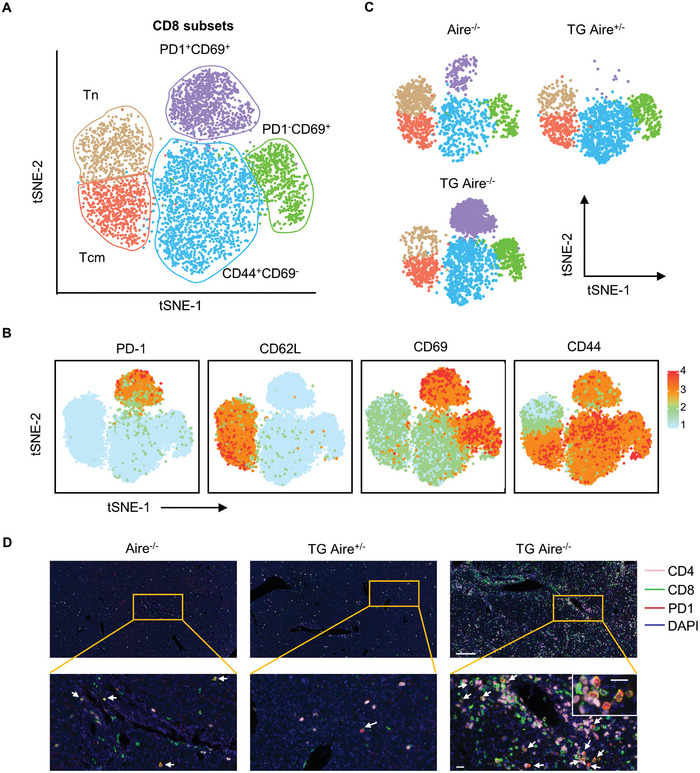
Enhanced PD‐1^+^CD8^+^ T cell response in the livers of dnTGFβRII Aire^−/‐^ mice. A) Merged t‐SNE plots of CD8^+^ T cells from 3 representative liver samples of Aire^−/−^, TG Aire^+/−^ and TG Aire^−/−^ mice. B) Expression distributions of PD‐1, CD62L, CD69, and CD44 on merged t‐SNE plots of CD8^+^ T cells. C) Split t‐SNE plots of (A). D) Immunohistochemistry CD4, CD8, and PD‐1 in the livers of Aire^−/−^, TG Aire^+/−^ and TG Aire^−/−^ mice. White arrows reflect PD‐1^+^CD8^+^ T cells. Scale bar in the upper row, 200 µm; scale bars in the bottom row, 20 µm. All results are reported from one representative experiment from at least three independent repeats.

We next analyzed the expression levels of PD‐1 as well as CD44, CD62L, and CD69 at the protein level by flow cytometry. Based on the expression levels of these surface markers, CD8^+^ T cells exhibited five distinct clusters in the livers of 2–4 week‐old Aire^−/−^, dnTGFβRII Aire^+/−^ and dnTGFβRII Aire^−/−^ mice (**Figure** **3**A,B), including naïve T cells (CD44^−^CD62L^+^ Tn), central memory T cells (CD44^+^CD62L^+^ Tcm), CD69^+^PD‐1^+^, PD‐1^−^CD69^+^ and CD44^+^CD69^−^ T cells. The split t‐SNE plots demonstrated that the massively expanded CD8^+^ T cells expressed CD69^+^PD‐1^+^ in the livers of dnTGFβRII Aire^−/−^ mice (Figure 3C). We further detected the expressions of PD‐1 and CD8 in the livers using multiplex immunohistochemistry, which also revealed the expansion of PD‐1^+^CD8^+^ T cells in the livers of dnTGFβRII Aire^−/−^ mice, and showed their infiltration in the portal areas and lobules (Figure 3D). Very few PD‐1^+^CD8^+^ T cells were detected in the spleen, blood or thymus of dnTGFβRII Aire^−/−^ mice using flow cytometry (Figure S3A,B, Supporting Information). Next, we sorted the hepatic PD‐1^+^ and PD‐1^−^CD8^+^ T cells from the livers of dnTGFβRII Aire^−/−^ mice and adoptively transferred them into the Rag1^−/−^ mice. After 12 weeks, we detected the frequencies of PD‐1^+^CD8^+^ and PD‐1^−^CD8^+^ T cells in lymphocytes isolated from multiple organs (Figure S4A, Supporting Information). These results demonstrated a high proportion of transferred PD‐1^+^CD8^+^ T cells in the livers of recipients, but low proportions in other tissues. However, the transferred PD‐1^−^CD8^+^ T cells exhibited comparable proportions in the blood, spleen and liver (Figure S4B, Supporting Information). Interestingly, we found there were ≈30% of PD‐1^+^CD8^+^ T cells exist in the livers of recipient mice which received donor PD‐1^−^CD8^+^ T cells, whereas only low frequencies of PD‐1^+^CD8^+^ T cells were found in the spleen and blood (Figure S4C,D, Supporting Information). These results indicate that PD‐1^+^CD8^+^ T cells represent a specifically expanded subset in the livers of dnTGFβRII Aire^−/−^ mice.

### 2.4 PD‐1^+^CD8^+^ T Cells Exhibit High Activation and Cytotoxicity

**Figure 4:**
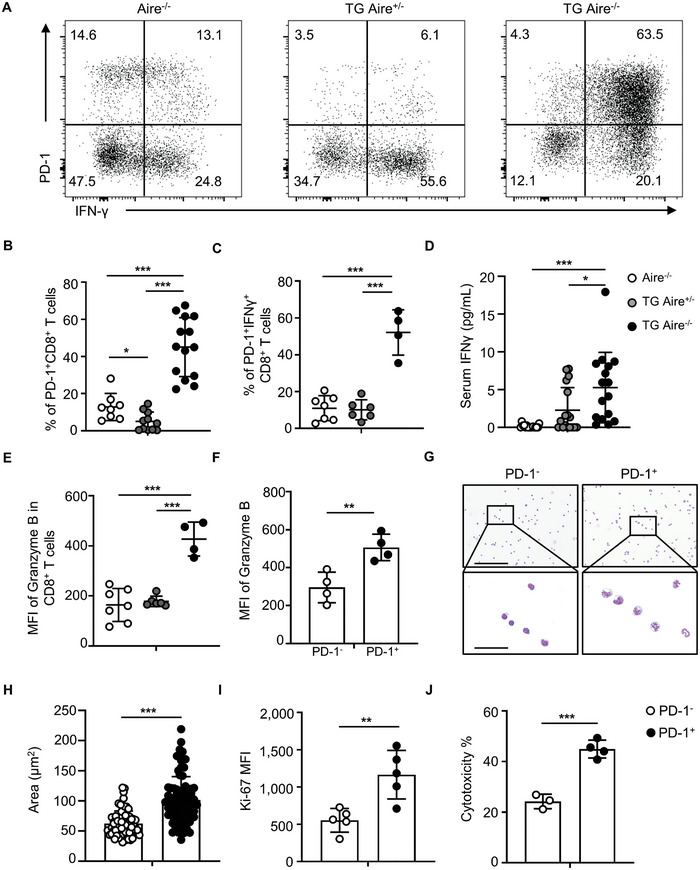
Functional activation of PD‐1^+^CD8^+^ T cells in the livers of dnTGFβRII Aire^−/−^ mice. A) Representative flow cytometry results of PD‐1 and IFNγ expression by the gated population of CD3^+^CD8^+^ cells in the livers of Aire^−/−^, TG Aire^+/−^ and TG Aire^−/−^ mice. Statistical analysis of the percentage of B) PD‐1^+^CD8^+^ T cells and C) PD‐1^+^CD8^+^IFNγ^+^ T cells in the livers of Aire^−/−^ (n = 8 or 7), TG Aire^+/−^ (n = 11 or 6) and TG Aire^−/−^ mice (n = 14 or 4). D) Serum levels of IFNγ in Aire^−/−^ (n = 16), TG Aire^+/−^ (n = 17) and TG Aire^−/−^ (n = 16) mice. E) Statistical analysis of the MFI of granzyme B in CD8^+^ T cells from Aire^−/−^ (n = 7), TG Aire^+/−^ (n = 6) and TG Aire^−/−^ (n = 4) mice. F) Statistical analysis of the MFI of granzyme B in PD‐1^+^CD8^+^ and PD‐1^−^CD8^+^ T cells of TG Aire^−/−^ (n = 4) mice. G) Giemsa staining results of PD‐1^+^CD8^+^ and PD‐1^−^CD8^+^ T cells. Scale bar in the upper row, 250 µm; scale bar in the bottom row, 50 µm. H) Statistical analysis of the area of PD‐1^+^CD8^+^ and PD‐1^−^CD8^+^ T cells in one 200 × field. I) Statistical analysis of the expression level of Ki‐67 in PD‐1^+^CD8^+^ and PD‐1^−^CD8^+^ T cells from livers of TG Aire^−/−^ mice (n = 5) by flow cytometry. J) Statistical analysis of the cytotoxicity of 1 × 10^5^ PD‐1^+^CD8^+^ or PD‐1^−^CD8^+^ T cells following co‐culture with 1 × 10^4^ isolated primary hepatocytes from wild‐type mice in vitro, detected the levels of lactate dehydrogenase (LDH) in supernatants and calculated the cytotoxicity percentages after 20 h. Data are means ± SD. *p < 0.05; **p < 0.01; ***p < 0.001, by one‐way ANOVA (B to E) or Student's *t* test (F, and H to J). All results are reported from one representative experiment from at least three independent repeats.

PD‐1^+^CD8^+^ T cells in dnTGFβRII Aire^−/−^ mice produce high levels of interferon‐γ (IFN‐γ) (**Figure** **4**A). Statistical analysis of the frequencies of the total PD‐1^+^CD8^+^ and the subset of PD‐1^+^IFN‐γ^+^CD8^+^ T cells demonstrated significant increases in dnTGFβRII Aire^−/−^ mice compared to dnTGFβRII Aire^+/−^ and Aire^−/−^ control groups (Figure 4B,C). Furthermore, we detected higher levels of IFN‐γ in the serum of dnTGFβRII Aire^−/−^ mice (Figure 4D). The levels of granzyme B in hepatic CD8^+^ T cells of dnTGFβRII Aire^−/−^ mice were higher than that of dnTGFβRII Aire^+/−^ and Aire^−/−^ mice (Figure 4E), and PD‐1^+^CD8^+^ T cells had significantly higher levels of granzyme B than PD‐1^−^CD8^+^ T cells (Figure 4F). The sizes of PD‐1^+^CD8^+^ T cells were larger than that of PD‐1^−^CD8^+^ T cells (Figure 4G,H). PD‐1^+^CD8^+^ T cells expressed higher levels of Ki‐67 than PD‐1^−^CD8^+^ T cells (Figure 4I). Co‐culture with primary hepatocytes indicated significantly higher cytotoxicity mediated by PD‐1^+^CD8^+^ T cells compared to PD‐1^−^CD8^+^ T cells (Figure 4J). To investigate the presence and functional differences among different PD‐1^+^CD8^+^ T cell subpopulations, we simultaneously labeled lymphocytes in the livers of dnTGFβRII Aire^−/−^ mice with antibodies against PD‐1 and Tim3. The results revealed two groups of PD‐1^+^ cells with differing levels of Tim3 expression (Figure S5A, Supporting Information). Notably, PD‐1^+^Tim3^+^ T cells exhibited significantly higher levels of Ki‐67 compared to PD‐1^+^Tim3^−^ cells (Figure S5B, Supporting Information). Additionally, co‐culture experiments with primary hepatocytes demonstrated that both PD‐1^+^Tim3^−^ and PD‐1^+^Tim3^+^ T cells displayed strong cytotoxic effects against target cells, with comparable killing capacities (Figure S5C, Supporting Information). These results indicate that the expanded PD‐1^+^CD8^+^ T cells in the livers of dnTGFβRII Aire^−/−^ mice exhibit strong functional activity and cytotoxic capability.

### 2.5 PD‐1^+^CD8^+^ T Cells are the Pathogenic Cell Subset in dnTGFβRII Aire^−/−^ Mice

**Figure 5:**
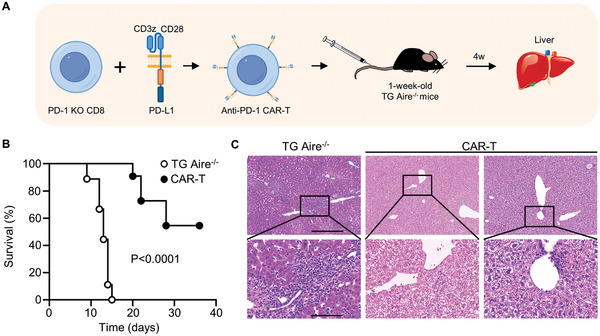
PD‐1^+^CD8^+^ T cell is the pathogenic cells of dnTGFβRII Aire^−/‐^ mice. A) Schematic diagram shows the PD‐1^+^ cells depletion assay. 1‐week‐old TG Aire^−/‐^ mice were intravenously injected with 5 × 10^5^ anti‐PD‐1 CAR‐T cells and monitored the diseases. B) Survival curves of TG Aire^−/−^ mice treated in vivo with (n = 11) or without (n = 9) anti‐PD‐1 CAR‐T cells. C) Liver histology results of anti‐PD‐1 CAR‐T cells treated (5‐week‐old) or untreated (2‐week‐old) TG Aire^−/−^ mice. Scale bar in the upper row, 400 µm; scale bar in the bottom row, 100 µm. All results are reported from one representative experiment from at least three independent repeats.

To further explore the role of PD‐1^+^CD8^+^ T cells in the development of autoimmune liver diseases in dnTGFβRII Aire^−/−^ mice, we designed CAR‐T cells, which specifically targeting PD‐1^+^ cells and conducted in vivo experiments to deplete PD‐1^+^ cells in the model mice (**Figure** **5**A). The flow cytometry results showed a significantly decreased frequency of PD‐1^+^CD8^+^ T cells in the livers of CAR‐T treated dnTGFβRII Aire^−/−^ mice compared to those without CAR‐T treatment (Figure S6A,B, Supporting Information). This demonstrated the effective clearance capability of CAR‐T on PD‐1^+^CD8^+^ T cells in liver. We found that the clearance of PD‐1^+^ cells effectively improved their survival status (Figure 5B) and significantly alleviated pathological damage and inflammatory infiltration in the liver (Figure 5C). These findings confirm the crucial pathogenic role of PD‐1^+^CD8^+^ T cells in dnTGFβRII Aire^−/−^ mice.

### 2.6 PD‐1^+^CD8^+^ T Cells Promote Disease Progression by Inducing GSDMD‐Mediated Pyroptosis in Hepatocytes

**Figure 6:**
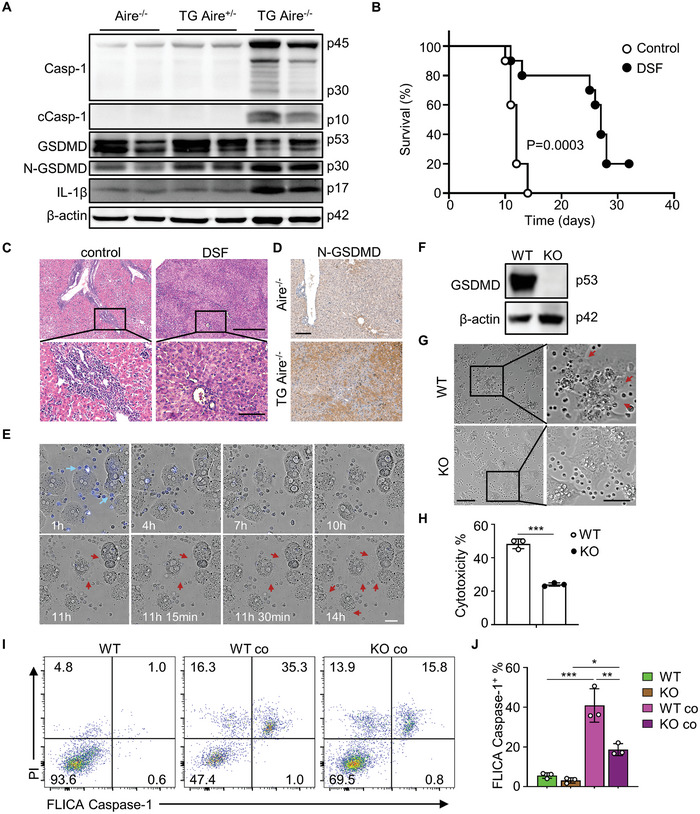
PD‐1^+^CD8^+^ T cells from dnTGFβRII Aire^−/−^ mice induce GSDMD mediated pyroptosis of hepatocytes. A) Detection of the expression levels of GSDMD, caspase‐1 and IL‐1β in the livers of Aire^−/−^, TG Aire^+/−^ and TG Aire^−/−^ mice by Western blot using anti‐GSDMD antibody (sc‐393581), anti‐caspase‐1 antibody (ab179515) and anti‐IL‐1β antibody (ab9722). B) Survival curves of TG Aire^−/−^ mice treated in vivo with (n = 10) or without (n = 10) 50mg kg^−1^ disulfiram (DSF) by intraperitoneal injection every day from 7 days of age. C) Liver histology results of DSF treated (4‐week‐old) or untreated (about 2‐week‐old) TG Aire^−/−^ mice. Scale bar in the upper row, 500 µm; scale bar in the bottom row, 100 µm. D) Immunohistochemistry results of the expressions of N‐GSDMD in the liver tissues from Aire^−/−^ and TG Aire^−/−^ mice. Scale bar, 100 µm. E) 1 × 10^5^ PD‐1^+^CD8^+^ T cells were co‐cultured with 1 × 10^4^ isolated primary hepatocytes and the cell morphology was observed by high content imaging system. Blue arrows reflect PD‐1^+^CD8^+^ T cells and red arrows demonstrate hepatocytes in the process of pyroptosis. Scale bar, 50 µm. F) Detection of the expression levels of GSDMD in AML12 cells with/without GSDMD knockout by Western blot using anti‐GSDMD antibody (ab219800). G) 1 × 10^5^ CD8^+^ T cells isolated from the livers of dnTGFβRII Aire^−/−^ mice were co‐cultured with 1 × 10^4^ AML12 cells with/without GSDMD knockout and the cell morphology was imaged using a confocal microscope after 24 h. Scale bar in the left column, 100 µm; scale bar in the right column, 50 µm. H) The cell culture supernatants from (G) were detected the LDH levels and analyzed for cytotoxicity at 24 h. I) The cultured AML12 cells in (G) were labeled with FAM‐FLICA caspase‐1 and PI after 24h, then the cells were digested and detected the fluorescence signal using flow cytometry. Flow cytometry results show the fluorescence signal intensity of FLICA caspase‐1 and PI. WT co and KO co refer to wild type and GSDMD knockout AML12 cells which co‐cultured with PD‐1^+^CD8^+^ T cells respectively. J) Statistical analysis of the percentage of FLICA caspase‐1^+^ AML12 in total AML12 cells. Data are means ± SD. *p < 0.05; **p < 0.01; ***p < 0.001, by log‐rank survival analysis (B) or unpaired Student's t test (H) or one‐way ANOVA (J).

Herein we initially assessed the expression levels of pyroptosis‐related proteins in the livers of dnTGFβRII Aire^−/−^ and control mice. The results demonstrated that the levels of N‐GSDMD, cleaved caspase‐1 and IL‐1β in the livers of dnTGFβRII Aire^−/−^ mice were significantly higher than those in control mice (**Figure** **6**A), while the cleavage levels of GSDME and caspase‐3 had no significant differences (Figure S7A, Supporting Information), consistent with the activation of the GSDMD mediated pyroptosis pathway. In addition, the expression levels of NLRP3 (NOD‐like receptor protein 3) in the livers of dnTGFβRII Aire^−/−^ mice showed significant upregulation compared to control mice (Figure S7B, Supporting Information). Subsequently, we administered an inhibitor disulfiram (DSF), which reported to be a specific inhibitor of GSDMD‐mediated pore formation.^[^ ^9^ ^]^ The results demonstrate that treatment of DSF significantly improved the survival (Figure 6B), and a notable alleviation of immunpathology (Figure 6C). Subsequently, we carried out immunohistochemistry to detect the expression of N‐GSDMD in liver. Compared to the Aire^−/−^ control mice, the levels of N‐GSDMD were significantly higher in dnTGFβRII Aire^−/−^ mice (Figure 6D). To directly investigate whether PD‐1^+^CD8^+^ T cells can induce pyroptosis in hepatocytes, we sorted PD‐1^+^CD8^+^ T cells from the livers of dnTGFβRII Aire^−/−^ mice and isolated the primary hepatocytes from wild‐type mice for in vitro co‐culture. Using a high‐content cell imaging system, we observed that PD‐1^+^CD8^+^ T cells could induce obvious morphological change of pyroptosis in hepatocytes (Figure 6E; Movie S1, Supporting Information). We further performed FAM‐FLICA caspase‐1 and propidium iodide (PI) dual‐staining assay, the confocal fluorescence imaging results clearly showed that many primary hepatocytes exhibiting typical characteristics of pyroptosis when co‐cultured with PD‐1^+^CD8^+^ T cells, exhibited strong fluorescence for FLICA caspase‐1. In contrast, such phenomena were not observed in hepatocytes that cultured alone (Figure S8A, Supporting Information). Moreover, the detection of LDH in the culture supernatant also indicated that co‐culture with PD‐1^+^CD8^+^ T cells significantly induced a higher mortality rate of hepatocytes (Figure S8B, Supporting Information). To further demonstrate that the cytotoxicity of hepatic PD‐1^+^CD8^+^ T cell in dnTGFβRII Aire^−/−^ mice is dependent on the GSDMD‐mediated pyroptosis pathway in hepatocytes, we generated GSDMD knockout AML12 cells (Figure 6F) and co‐cultured with PD‐1^+^CD8^+^ T cells isolated from the livers of dnTGFβRII Aire^−/−^ mice. The results revealed that the knockout of GSDMD effectively inhibited the occurrence of PD‐1^+^CD8^+^ T cell‐induced pyroptosis in AML12 cells (Figure 6G). Compared to control cells without GSDMD knockout, the cytotoxicity of CD8^+^ T cells against GSDMD knockout AML12 was markedly reduced (Figure 6H). Moreover, the percentage of FLICA caspase‐1^+^ AML12 cells significantly increased when co‐cultured with PD‐1^+^CD8^+^ T cells compared to AML12 cells cultured alone. In contrast, knockout of GSDMD in AML12 cells dramatically inhibited PD‐1^+^CD8^+^ T induced activation of caspase‐1 in AML12 cells (Figure 6I,J). Additionally, we found that the percentage of FLICA caspase‐1^+^ AML12 cells was significantly higher than the percentage of PI^+^ FLICA caspase‐1^−^ AML12 cells in co‐cultured AML12 cells (Figure S9A,B, Supporting Information). We further explored whether the depletion of PD‐1^+^ cells in dnTGFβRII Aire^−/−^ mice could inhibit the pyroptosis of hepatocytes using PD‐1 targeting CAR‐T cells. The immunohistochemical results indicated that CAR‐T treatment dramatically reduced N‐GSDMD level in the livers of dnTGFβRII Aire^−/−^ mice (Figure S10A, Supporting Information). The analysis results of RNA‐seq data revealed the overall gene expression pattern in the livers of dnTGFβRII Aire^−/−^ mice with CAR‐T cells treatment shows large difference compared to untreated dnTGFβRII Aire^−/−^ mice, while being closer to that of wild‐type (WT) mice (Figure S10B, Supporting Information). Additionally, the enrichment levels of pyroptosis‐related pathways and the expression levels of pyroptosis‐related genes in the livers of CAR‐T treated dnTGFβRII Aire^−/−^ mice significantly decreased compared to those without CAR‐T treatment, restoring levels more similar to those of WT mice (Figure S10C,D, Supporting Information). These results suggest that hepatic PD‐1^+^CD8^+^ T cells in dnTGFβRII Aire^−/−^ mice can promote disease progression by inducing GSDMD mediated pyroptosis in hepatocytes.

### 2.7 PD‐1^+^CD8^+^ T Cells Mediate Pyroptosis in Target Cells through the Production of Granzyme B and Perforin‐1

**Figure 7:**
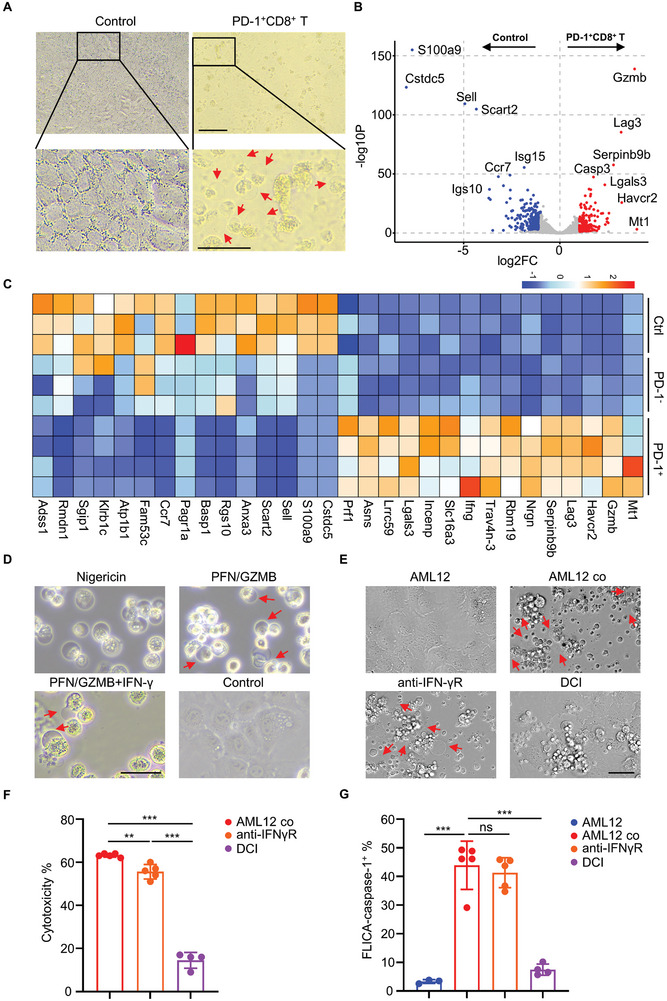
Perforin‐1 and granzyme B produced by PD‐1^+^CD8^+^ T cells of dnTGFβRII Aire^−/‐^ mice mediate hepatocyte pyroptosis. A) A transwell assay is used to detect non‐contact function of PD‐1^+^CD8^+^ T cells and AML12 cells. 1 × 10^6^ PD‐1^+^CD8^+^ T cells sorted from the livers of dnTGFβRII Aire^−/−^ mice were added to the upper layer 24‐well insert with 0.4 µm membrane pore, and 5 × 10^5^ AML12 cells were added to the bottom, after 72h, the cell morphology of AML12 in the center of bottom layer were imaged. The controls in the upper chambers were supplemented with hepatic CD8^+^ T cells from dnTGFβRII Aire^+/−^ mice for comparison. Scale bar in the upper row, 100 µm; scale bar in the bottom row, 50 µm. B) 3 × 10^5^ PD‐1^+^CD8^+^ T, PD‐1^−^CD8^+^ T cells sorted from the livers of dnTGFβRII Aire^−/−^ mice or hepatic CD8^+^ T cells from dnTGFβRII Aire^+/−^ mice were cultured in vitro with serum‐free culture medium. After 24 h of culture, the culture supernatants were collected for proteomic analysis. The volcano plot displays the differential protein expression in supernatant of CD8^+^ T cells from dnTGFβRII Aire^+/−^ mice. C) The heat map results exhibit the relative levels of differential proteins in the culture supernatants between PD‐1^+^CD8^+^ T cells and control groups from (B). D) AML12 cells treated with 10 µM nigericin or sublytic concentration of perforin‐1 and 500 nM granzyme B, or pretreated with 100 ng mL^−1^ IFN‐γ for 24 h then treated with perforin‐1 and granzyme B and imaging after 18 h in vitro cultured. AML12 cultured alone group was used as control group. Scale bar, 40 µm. E) 1 × 10^5^ PD‐1^+^CD8^+^ T cells were co‐cultured with 1 × 10^4^ AML12 cells treated with/without 50 µg mL^−1^ anti‐IFN‐γR antibody or pretreated the PD‐1^+^CD8^+^ T cells with 100 µM DCI for 1 h before co‐cultured with AML12 cells. AML12 cultured alone group was used as control group. The cell morphology imaged under a confocal microscopy after 24 hr. Scale bar, 50 µm. F) The cell culture supernatants from (E) were detected the LDH levels and analyzed for cytotoxicity at 24 h. G) The cultured AML12 cells in (E) were labeled with FAM‐FLICA caspase‐1 and PI after 24h, then the cells were digested and detected the fluorescence signal using flow cytometry. Statistical analysis of the percentage of FLICA caspase‐1^+^ AML12 in total AML12 cells. AML12 co in (E), (F), and (G) refer to AML12 cells co‐cultured with PD‐1^+^CD8^+^ T cells. Red arrows in (A), (D), and (E) show AML12 cells note the process of pyroptosis. Data are means ± SD. A non‐parameteric Wilcoxon rank sum test was performed to assess the statistical significance in (B). **p < 0.01; ***p < 0.001, by one‐way ANOVA (F and G).

We utilized transwell experiments to investigate whether PD‐1^+^CD8^+^ T cell‐mediated pyroptosis in hepatocytes depends on direct contact. PD‐1^+^CD8^+^ T cells in the upper chamber induced pyroptosis in AML12 cells without direct contact (**Figure** **7**A). This indicated that PD‐1^+^CD8^+^ T cells can induce pyroptosis in target cells through the secretion of active molecules. To identify the components of these molecules, we then performed proteomic analysis on the culture supernatants of hepatic PD‐1^+^CD8^+^ cells and PD‐1^−^CD8^+^ T cells from dnTGFβRII Aire^−/−^ mice after culture for 24 h, as well as the culture supernatant of hepatic CD8^+^ T cells from dnTGFβRII Aire^+/−^ mice. The volcano plot results showed a significant increase of granzyme B in the supernatant of PD‐1^+^CD8^+^ T cells compared to that of CD8^+^ T cells from dnTGFβRII Aire^+/−^ mice (Figure 7B). The heatmap results also demonstrated an increase of granzyme B, as well as IFN‐γ and perforin‐1 in the supernatant of PD‐1^+^CD8^+^ T cells compared to PD‐1^−^CD8^+^ cells and CD8^+^ T cells from the dnTGFβRII Aire^+/−^ mice (Figure 7C).

To confirm whether granzyme B and perforin‐1 can induce pyroptosis in hepatocytes, we added recombinantly expressed granzyme B and perforin‐1 to the medium of in vitro cultured AML12 cells. The results indicate noticeable pyroptosis in AML12 cells was observed. Pre‐treatment with IFN‐γ did not significantly affect the occurrence of pyroptosis (Figure 7D). This result indicated that PD‐1^+^CD8^+^ T cells may induce pyroptosis in hepatocytes by producing high levels of granzyme B and perforin‐1. Subsequently, we pretreated PD‐1^+^CD8^+^ T cells with DCI (3, 4‐dichloroisocoumarin), an inhibitor of granzymes, or blocking antibodies against IFN‐γ receptors into the in vitro co‐culture system of PD‐1^+^CD8^+^ T cells and AML12 cells. DCI pretreatment significantly suppressed pyroptosis in AML12 cells (Figure 7E), and the levels of LDH release also decreased significantly (Figure 7F), as well as the percentage of FLICA caspase‐1^+^ AML12 was dramatically reduced (Figure 7G). Treatment with blocking antibodies against IFN‐γ receptors had no effect on pyroptosis of target cells (Figure 7E‐G). These results demonstrate that PD‐1^+^CD8^+^ T cells mediate pyroptosis in target cells through the production of granzymes. This is consistent with the results of proteomic analysis and in vitro experiments with recombinant proteins, indicating that PD‐1^+^CD8^+^ T cells induces pyroptosis in target cells by producing grazyme B and perforin‐1.

## 3 Discussion

Our previous work has reported a critical role of autoreactive CD8^+^ T cells in the pathogenesis of autoimmune cholangitis.^[^ ^8^, ^10^ ^]^ However, it remains unclear whether there are specific pathogenic subsets within CD8^+^ T cells and the molecular mechanisms by which they mediate the development of pathology. PD‐1 is primarily expressed on the surface of activated T cells, and is an important regulator of immune homeostasis.^[^ ^11^ ^]^ The absence of PD‐1 can lead to the development of autoimmune disease; blockade of the PD‐1 checkpoint exacerbates autoimmune diseases in both human and mouse models.^[^ ^12^ ^]^ However, depletion of PD‐1^+^ cells ameliorates autoimmune murine models of diabetes and encephalomyelitis.^[^ ^12b^ ^]^ Herein we demonstrate the clonal expansion of functionally activated PD‐1^+^CD8^+^ T cells in the livers of dnTGFβRII Aire^−/−^ mice, which play a pathogenic role in the progression of disease.

Consistent with our findings, clonally expanded effector PD‐1^+^CD8^+^ T cells have been identified in other autoimmune disease. Femke et al. reported that the presence of PD‐1^+^CD8^+^ T cells in the synovial fluid of patients with juvenile idiopathic arthritis. While these PD‐1^+^CD8^+^ T cells express high levels of other immune checkpoint molecules such as *Havcr2* and *Lag3*, the authors report that the gene transcription pattern of PD‐1^+^CD8^+^ T cells is similar to that of effector memory T and tissue‐resident memory T cells. Moreover, the elevated secretion of pro‐inflammatory cytokines and the clonal expansion indicate they are activated effector cells rather than functionally exhausted cells.^[^ ^13^ ^]^ This suggests that PD‐1^+^CD8^+^ T cells with high expression of immune checkpoints may exhibit distinctly different functional states due to differences in the environment between tumors and autoimmune diseases. In at least some autoimmune diseases, the significantly expanded PD‐1^+^CD8^+^ T cell population with high expression of inflammatory cytokines may represent a highly activated pathogenic cell subset. There are few reports on the role of PD‐1^+^CD8^+^ T cells in human liver autoimmunity. A recent study reported an overexpression of immune checkpoint molecules in the liver of AIH, accompanied by a high proportion of PD‐1^+^CD8^+^ T cells. Moreover, there was a positive correlation between the abundance of PD‐1^+^CD8^+^ T cells and the severity of AIH (reflected in AST and ALT levels).^[^ ^5^ ^]^ Consistent with the findings of this study, our results demonstrate a significant increase in a subset of PD‐1^+^CD8^+^ T cells in the liver. These cells exhibit clonal expansion, strong functional activity, and cytotoxicity against hepatocyte. Importantly our data herein elucidates their role in promoting disease progression and the molecular mechanisms involved. This provides an attractive approach to investigate this population as effective therapeutic targets.

In addition to the specific expansion of CD8^+^ T cell clones found in the dnTGFβRII Aire^−/−^ mouse, we also observe a clonal expansion of clonotype 2 in the CD8^+^ T cells of dnTGFβRII mice. We assume that due to the absence of peripheral TGF‐β signaling in dnTGFβRII mice, which allows escaped autoreactive T cells to undergo significant clonal expansion when stimulated by peripheral self‐antigens. In contrast, in dnTGFβRII Aire^+/−^ and dnTGFβRII Aire^−/−^ mice, the partial or complete Aire gene defect may permit more different autoreactive T cell clones to enter the periphery and expand, leading to fewer or undetectable clonotype 2 in these mice. This highlights the critical role of peripheral tolerance in regulating the generation and activation of autoreactive T cells.

Our adoptively transfer experiments with Rag1^−/−^ mice show that approximately 30% of the PD1^−^CD8^+^ T cells that enter the recipient mice livers differentiate into PD1^+^CD8^+^ T cells, indicating the potential for PD1^−^CD8^+^ T cells to transition into the PD1^+^CD8^+^ T cells. However, the proportions of PD1^+^CD8^+^ T cells in tissues such as the thymus, peripheral blood, and spleen are very low. This suggests that self‐reactive CD8^+^ T cells, which persist due to deficiencies in the Aire gene, predominantly exhibit a PD1^−^CD8^+^ T cell phenotype during early thymic development and upon entering the peripheral circulation. Only after entering the liver and receiving further stimulation, some of these PD1^−^CD8^+^ T cells differentiate into PD1^+^CD8^+^ T cells, acquiring enhanced functional activity and proliferative capacity. However, further research is needed to explore in detail whether specific PD1^−^CD8^+^ T cell subpopulations are required for differentiation into PD1^+^CD8^+^ T cells. In addition, PD1^+^CD8^+^ T cells highly express the tissue‐resident marker CD69 while downregulating the migration‐related molecule CCR7, suggest that PD1^+^CD8^+^ T cells differentiated in the liver may acquire tissue residency capabilities, facilitating their accumulation in the liver. In contrast, PD1^−^CD8^+^ T cells exhibit lower levels of CD69 and higher levels of CCR7, facilitating their circulation and exchange between different tissues. Although transfer experiments indicate that PD‐1^+^CD8^+^ T cells tend to preferentially differentiate and survive in the liver. However, at the three‐month endpoint, we did not observe significant pathological damage or inflammatory infiltration in the livers of Rag1^−/−^ recipient mice. We believe this may be due to an insufficient number of transferred cells. Although PD‐1^+^ CD8^+^ T cells exhibit stronger cytotoxic functions compared to PD‐1^−^ cells, they also show a higher rate of cell death (data not shown). Actually, we detected the absolute number of PD‐1^+^CD8^+^ T cells present in the livers of the recipient mice after three months was comparable to the initial number transferred (data not shown), resulting in minimal observable inflammatory infiltration and damage in the livers. To address this issue, we further confirmed the pathogenic role of these cells by isolating primary mouse hepatocytes and co‐culturing them with PD‐1^+^CD8^+^ T cells.

Our scRNA‐seq data indicates the presence of two distinct *Pdcd1* ^+^CD8^+^ T cell populations. Through further differential gene and functional analysis, we found that the T10_CD8_Havcr2 cluster exhibits a stronger proliferation capacity and expresses higher levels of *Havcr2* compared to the T07_CD8_Pdcd1 cluster. Flow cytometry analysis of Tim3 and Ki‐67 confirms that the PD‐1^+^Tim3^+^ cells have higher levels of Ki‐67 and Tim3 expression, likely corresponding to T10_CD8_Havcr2, while the PD‐1^+^Tim3^−^ cluster is more similar to T07_CD8_Pdcd1. Furthermore, co‐culture experiments with primary hepatocytes show that both populations exhibited strong cytotoxicity against target cells, with comparable killing ability. Therefore, we believe that both of these cell populations exhibit strong effector functions. In all subsequent analyses and experiments, we treated these two cell populations as a unified PD‐1^+^CD8^+^ T cell subset.

We further explore the molecular mechanisms by which PD‐1^+^CD8^+^ T cells promote autoimmunity. Through RNA‐seq of liver tissues and Western blot analysis, we observe a significant activation of the liver pyroptosis pathway. Therefore, we hypothesize that PD‐1^+^CD8^+^ cells influence the progression of the disease by inducing pyroptosis in target cells. Through in vitro co‐culture experiments, we demonstrate that PD‐1^+^CD8^+^ T cells can induce pyroptosis in primary hepatocytes. There is other data on pyroptosis that should be discussed. For example, the release of IL‐1β, IL‐18, and HMGB1 from monocyte/macrophage pyroptosis exacerbates systemic lupus erythematosus.^[^ ^14^ ^]^ Vakrakou et al. reported that cf‐DNA can promote macrophage pyroptosis via overactivated NLRP3 inflammasome in Sjögren's syndrome.^[^ ^15^ ^]^ Several studies have demonstrated that pyroptosis may play a major role in the development of non‐alcoholic steatohepatitis (NASH).^[^ ^16^ ^]^ In addition, inflammasome‐mediated pyroptosis is critical for the inflammatory response and severity of liver injury.^[^ ^17^ ^]^ In ConA treated mice, the levels of NLRP3, Cleaved caspase‐1, and IL‐1β in the livers were significantly elevated, and there was a pyroptosis. In contrast, NLRP3^−/−^ and caspase‐1^−/−^ mice demonstrate suppressed development of autoimmune hepatitis, with a reduction in liver injury, ALT levels, and pyroptosis.^[^ ^18^ ^]^ NLRP3 activation correlates with disease activity in patients with PBC.^[^ ^19^ ^]^ Our data showing that autoreactive CD8^+^ T cells induce hepatocyte pyroptosis has implications that extend beyond the murine model used in this study.

CAR‐T therapy has demonstrated significant potential in the clinical treatment of tumors.^[^ ^20^ ^]^ CAR‐T cells targeting CD19 and B‐cell maturation antigen (BCMA) have shown remarkable and persistent therapeutic effects in refractory patients with B‐cell and plasma cell malignancies, leading to FDA approval for the treatment of leukemia, lymphoma, and multiple myeloma.^[^ ^21^ ^]^ Recent studies suggest that CAR‐T therapy also exhibits notable therapeutic effects in various autoimmune diseases such as systemic lupus erythematosus (SLE) and systemic sclerosis, potentially serving as a treatment strategy for these diseases.^[^ ^20^, ^22^ ^]^ In this study, we reveal the pathogenic role of PD‐1^+^CD8^+^ T cells in a murine model of autoimmune liver disease by inducing pyroptosis in target cells. While CAR‐T treatment significantly eliminate PD‐1^+^CD8^+^ T cells in the livers of dnTGFβRII Aire^−/‐^ mice, it also reduce the levels of N‐GSDMD and inhibited the activation of pyroptosis‐related pathways, restoring them to levels comparable to those in control mice. These results indicate that the design of CAR‐T cells targeting PD‐1^+^ T cells holds promise for potential therapeutic effects in autoimmune liver diseases.

Pyroptosis includes the caspase‐1‐mediated classical pathway, Caspase‐4/5/11‐mediated non‐canonical pathway, Caspase‐3/8‐mediated pathway, and Granzyme‐mediated pathway.^[^ ^23^ ^]^ Our data suggests that direct cell‐to‐cell contact is not necessary for the induction of pyroptosis, suggesting that PD‐1^+^CD8^+^ T cells may induce pyroptosis through protein secretion. Proteomic analysis reveal a significant increase in granzyme B in the culture supernatant of PD‐1^+^CD8^+^ T cells, while differences in other granzymes are relatively minor. To explore the role of granzyme B in target cell pyroptosis, we treated the co‐cultured cells with DCI, a widely referenced pan‐granzyme inhibitor.^[^ ^23^, ^24^ ^]^ The results demonstrate that PD‐1^+^CD8^+^ T cell‐induced hepatocyte death is dependent on granzymes, and DCI pre‐treatment significantly reduce the proportion of FLICA caspase‐1^+^ target cells in the co‐culture system, indicating that granzymes produced by PD‐1^+^CD8^+^ T cells plays a crucial role in the activation of caspase‐1 in target cells. Additionally, treating AML12 cells with perforin‐1 and recombinant granzyme B in vitro indeed induce hepatocyte pyroptosis. Therefore, we believe these results collectively indicate that PD‐1^+^CD8^+^ T cells can promote caspase‐1 activation and induce pyroptosis in target cells through the production of granzyme B. This provides a new understanding of the pathogenesis of autoimmune liver disease.

Inflammatory diseases are often accompanied by cell damage or death, leading to the production of DAMPs (damage‐associated molecular patterns), which promote the progression and maintenance of inflammation through various mechanisms.^[^ ^25^ ^]^ In our mouse model, massive cell death induced by perforin and granzyme B produced by PD‐1^+^CD8^+^ T cells is believed to result in the release of large amounts of DAMP molecules.^[^ ^26^ ^]^ On one hand, DAMPs can bind to TLRs expressed by hepatocytes to activate TLR signaling pathways,^[^ ^27^ ^]^ further promoting NF‐κB transcriptional activity, which was reported to regulate the caspase‐1 expression.^[^ ^28^ ^]^ On the other hand, DAMP signaling can also activate NLRP3 inflammasome, which cleave caspase‐1 and promote pyroptosis.^[^ ^25^, ^29^ ^]^ This is consistent with our findings of increased expression and activation levels of caspase‐1, as well as elevated levels of NLRP3, in the livers of model mice. Additionally, although granzyme B are generally believed to induce apoptosis by activating caspase‐3 or its substrates,^[^ ^30^ ^]^ an increasing number of studies have reported the important role of granzymes in inducing pyroptosis in target cells by directly cleaving various gasdermins.^[^ ^23^, ^31^ ^]^ Our western blot results indicate a significant elevation in the levels of N‐GSDMD in the livers, while the levels of N‐GSDME are unchanged. Moreover, the use of a specific inhibitor targeting GSDMD effectively mitigate the progression of disease. These indicates a novel mechanism that grazyme B derived from autoreactive CD8^+^ T cells may induce pyroptosis of hepatocytes by acting on GSDMD. Further research will be needed to directly confirm the potential cleavage data.

Although our findings indicate that PD‐1^+^CD8^+^T cells primarily induce pyroptosis in target cells, we do not exclude the potential role of granzyme B mediated apoptosis in our model mice. The results show that the co‐culture of PD‐1^+^CD8^+^ T cells and hepatocytes significantly increases the proportion of FLICA caspase‐1^+^PI^+^ cells, while also promoting a certain degree of increase in the proportion of FLICA caspase‐1^−^PI^+^ cells, compared to hepatocytes cultured alone. This suggests that while PD‐1^+^CD8^+^ T cells from dnTGFβRII Aire^−/−^ mice primarily exert their cytotoxic effects through the induction of hepatocyte pyroptosis, they may also operate via other non‐caspase‐1‐mediated forms of cell death. In fact, we observed an increase in apoptotic phenotypes among target cells under microscopy (data not shown). Therefore, we propose that in the livers of our model mice, PD‐1^+^CD8^+^ T cells can induce multiple cell death pathways in target cells, demonstrating potent cytotoxic effects, with pyroptosis being the primary mechanism. In addition, our results demonstrate DCI could effectively inhibit the cytotoxic effect of PD1^+^CD8^+^ T cells. However, even with DCI treatment, PD1^+^CD8^+^ T cells still exhibit approximately 15% cytotoxicity against target cells, indicating that they can also induce cell death through non‐granzymes‐dependent pathways. This may includes mechanisms such as FasL mediated Fas‐FasL signaling leading to cell death,^[^ ^32^ ^]^ as well as tumor necrosis factor‐α (TNF‐α) production activating the tumor necrosis factor receptor 1 (TNF‐R1) to trigger apoptotic or necrotic pathways in target cells.^[^ ^33^ ^]^

Our study found that pathogenic PD‐1^+^CD8^+^ T cells produce high levels of IFN‐γ, which reported to upregulate GSDMB expression in target cells and promote granzyme A induced pyroptosis, particularly in many GSDMB‐negative cell lines.^[^ ^23d^ ^]^ However, we found that IFN‐γ did not have a significant effect on the pyroptosis of hepatocytes induced by PD‐1^+^CD8^+^ T cells in our mouse model. Herein, we reveal that pyroptosis in hepatocytes primarily relies on the GSDMD‐mediated pathway. Therefore, we assume that IFN‐γ may strongly promote GSDMB expression without exerting a similar effect on GSDMD. Moreover, both primary hepatocytes and AML12 cells exhibit high baseline levels of GSDMD expression. Thus, even if IFN‐γ has a potential promoting effect on GSDMD expression, its impact on these cells may be limited, resulting in minimal effects on the pyroptosis levels of target cells.

It was reported that caspase‐1 primarily induces pyroptosis by cleaving GSDMD,^[^ ^6a^ ^]^ while caspase‐3 mainly promotes pyroptosis through the cleavage of GSDME.^[^ ^34^ ^]^ Our results show that the expression of caspase‐1 in the livers of our model mice is significantly elevated whereas there is no significant difference in caspase‐3 expression. This indicates that our model mice primarily induce pyroptosis via the caspase‐1/GSDMD pathway, independent of caspase‐3/GSDME. Research has found that during LPS‐induced pyroptosis in RAW264.7 mouse macrophages, the expression of GSDMD significantly increases, while GSDME expression decreases in the same cells.^[^ ^35^ ^]^ Consistent with this study, although we observed an increase in the activation levels of GSDMD in our model mice, the expression levels of GSDME were decreased. Such transcriptional regulation may enhance caspase‐1/GSDMD mediated pyroptosis in the context of inflammatory autoimmune diseases. Intervention therapy targeting GSDMD holds promise as a potential effective treatment. Disulfiram (DSF) is a FDA‐approved drug that has been used for decades to treat chronic alcohol addiction.^[^ ^36^ ^]^ Due to its confirmed safety, disulfiram is being repurposed, most notably for its role in regulating pathways and stress response through direct targeting of NPL4.^[^ ^37^ ^]^ In a recent study, disulfiram has been reported to be a specific inhibitor of GSDMD‐mediated pore formation, without affecting other GSDMs‐mediated pyroptosis pathways.^[^ ^9^ ^]^

**Figure 8:**
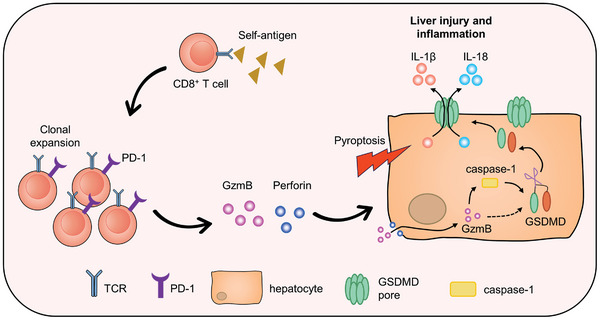
Schematic diagram of this research. Pathogenic PD‐1^+^CD8^+^ T cells in the livers of dnTGFβRII Aire^−/−^ mice exhibit strong functional activity and cytotoxicity against target cells. They produce high levels of granzyme B and perforin‐1, which further promotes the activation of caspase‐1 and cleavage of GSDMD, inducing the pyroptosis of hepatocytes and exacerbating liver injury and inflammation.

In conclusion, our data indicate that, in addition to traditional apoptosis, inducing target cell pyroptosis is important for CD8^+^ T cells to exert cytotoxic functions. However, there are no reported studies on the role of functionally activated CD8^+^ T cells inducing the pyroptosis of target cells in autoimmune disease and its impact on disease progression. Our results fill this research gap and suggest that auto‐reactive CD8^+^ T cells induce target cell pyroptosis through the production of granzyme B and perforin‐1, promoting the progression of diseases. Interestingly, in contrast to the reported mechanisms inducing pyroptosis in tumor cells,^[^ ^23c^ ^]^ we found that granzyme B may induce hepatocyte pyroptosis by acting on GSDMD but not GSDME (**Figure** **8**).

## 4 Experimental Section

### Mice

dnTGFβRII mice were derived from the vivarium of the University of California at Davis. Aire^−/−^ mice (B6.129S2‐AIREtm1.1Doi/J) were initially obtained from The Jackson Laboratory (Bar Harbor, ME, USA). The mice used herein were back‐crossed for 10 generations onto the C57BL/6 background. Male dnTGFβRII Aire^+/−^ mice were generated by intercrossing male dnTGFβRII mice with female Aire^+/−^ mice, then intercrossing with female Aire^+/−^ mice to generate dnTGFβRII Aire^−/−^ mice and control littermates. The mice used for this study were maintained in individually ventilated cages under specific pathogen‐free conditions in the Laboratory Animal Center, Guangdong Provincial People's Hospital. All mice were studied from 2–4 weeks of age regardless of gender unless mentioned otherwise. Animal experiments conformed to the guidelines outlined in the Guide for the Care and Use of Laboratory Animals, Guangdong Provincial People's Hospital.

Ethical approval for this study was obtained from the Ethics Committee of Guangdong Provincial People's Hospital (Approval No.: KY‐N‐2022‐086‐01).

### Histology

Liver sections were prepared and immediately fixed with 4% paraformaldehyde for 1–2 days. The tissues were embedded in paraffin and cut into 4‐µm slices. All slices were deparaffinized and stained with hematoxylin and eosin.

### Multicolor Immunohistochemistry and N‐GSDMD Immunohistochemical Staining

To detect PD‐1^+^CD8^+^ T cells and their spatial localization in the livers, multicolor immunohistochemical staining was performed. Briefly, liver paraffin sections were placed in an oven at 60 °C for 2 h, followed by deparaffinization and alcohol gradient rehydration in a series of 100%, 95%, 80%, and 70% alcohol. Antigen retrieval was first performed using sodium citrate buffer, then the sections were incubated with 3% methanol H_2_O_2_ for 10 min. Blocking was done with 10% goat serum at room temperature for 20 min. A 1:200 dilution of PD‐1 primary antibody (CST, 84 651) was applied, and the sections were incubated overnight at 4 °C in the dark. The next day, HRP‐conjugated secondary antibody was applied, followed by incubation at room temperature for 20 min. A 1:100 dilution of TSA fluorescent dye was added and incubated at room temperature for 10 min. A second round of antigen retrieval was performed using sodium citrate buffer, and the steps were repeated with a 1:200 dilution of CD8a (CST, 98 941), using a different TSA fluorescent dye. A third round of antigen retrieval was carried out using sodium citrate, followed by labeling with a 1:500 dilution of CD4 (Abcam, ab183685) and a different TSA fluorescent dye. Finally, DAPI (Beyotime, C1005) was added for nuclear staining at room temperature for 5 min. Imaging was performed using the Vectra Polaris Automated Quantitative Pathology Imaging System (Akoya Biosciences). To detect the expression levels of N‐GSDMD in liver tissues, single‐color immunohistochemical staining was performed. Similar to the multicolor immunohistochemistry procedure, after the blocking step, a 1:500 diluted primary N‐GSDMD (Affinity, DF13758) antibody was applied, followed by overnight incubation at 4 °C in the dark. The next day, HRP‐conjugated secondary antibody was applied, and the sections were incubated at room temperature for 20 min. DAB chromogenic solution was added and observed under a microscope until the desired color developed. The DAB reaction was then terminated, followed by hematoxylin counterstaining. The sections were rinsed in water to achieve blue color contrast, dehydrated through graded ethanol (80%, 95%, and 100%), and cleared in xylene before mounting. Finally, the slides were scanned for imaging.

### Flow Cytometry and Cell Sorting

For flow cytometry, 1 × 10^6^ mononuclear cells were incubated with an anti‐mouse FcR blocking reagent (BioLegend, San Diego, CA) at 4 °C for 15 min. Aliquots of the cells were then stained with a cocktail of fluorochrome‐conjugated monoclonal antibodies for 20 min at 4 °C that included anti‐CD44 FITC (IM7), anti‐NK1.1 PE‐Cy5 (PK136), anti‐CD279 PE‐CF594 (J43), anti‐CD8 Alexa700 (YTS156.7.7), anti‐CD4 BUV563 (GK1.5), anti‐CD62L BUV737 (MEL‐14), anti‐CD3 APC/Cy7 (17A2), anti‐CD45.2 V500, anti‐CD69 BV605 (H1.2F3) and anti‐CD19 BV 711 (6D5). Intracellular staining of Foxp3 was performed using a FOXP3 Fix/Perm Buffer protocol (BioLegend) and anti‐Foxp3 Alexa647 (MF‐14) antibody. To detect levels of intracellular cytokines, cells were re‐suspended in DMEM (high glucose) with 10% fetal bovine serum and stimulated with Cell Stimulation Cocktail (plus protein transport inhibitors) at 37 °C 5% CO_2_ for 4h. The cells were then stained with surface markers using anti‐CD45.2 APC‐Cy7, anti‐CD3 FITC (17A2), anti‐CD4 BV785 (RM4‐5), anti‐CD8β V500 (53‐6.7), anti‐CD69 BV421 (H1.2F3), anti‐PD‐1 PE‐CF594 (J43) and anti‐NK1.1 PE‐Cy5 (PK136), fixed with Fixation Buffer (BioLegend), and permeabilized with Permeabilization Wash Buffer (BioLegend). They were then stained for intracellular levels of IFN‐γ, granzyme B and TNF‐α using anti‐IFN‐γ PE‐Cy7 (XMG1.2), anti‐granzyme B PE (NGZB) and anti‐TNF‐α APC (MP6‐XT22) monoclonal antibodies, respectively. All antibodies were purchased from BioLegend (San Diego, CA, USA). The stained cells were washed with PBS (containing 0.2% BSA) and then re‐suspended in PBS and subjected to flow cytometric analysis using a LSRFortessa flow cytometry (BD Immunocytometry Systems, San Jose, CA, USA). Data obtained were analyzed with FlowJo software (Version 10.4, Tree Star, Inc, Ashland, USA). For select experiments, CD3^+^ CD4^−^ NK1.1^−^ CD8^+^ T cells were isolated from the livers of dnTGFβRII Aire^−/−^, dnTGFβRII Aire^+/−^ and Aire^−/−^ mice using a FACS Aria II cell sorter (BD Immunocytometry Systems, San Jose, CA, USA). The purity of the sorted cells was greater than 95%.

### T‐Distributed Stochastic Neighbor Embedding (t‐SNE) Analysis of Multi‐Parameter Flow Cytometry Data

For t‐SNE analysis of multi‐parameter flow cytometry data, the CD45^+^ lymphocytes or CD3^+^ T cells were gated using FlowJo software (Version 10.4, Tree Star, Inc, Ashland, USA), and exported the expression matrices of different samples from dnTGFβRII Aire^−/−^ mice, dnTGFβRII Aire^+/−^, dnTGFβRII and Aire^−/−^ mice. Next, 10^4^ cells were randomly selected from each matrices, to merge into one single matrix in R language (Version 3.5.3). Thence, this matrix was normalized by channel using a centered log‐ratio (CLR) transformation method embedded in NormalizeData function of Seurat package (Version 3.1.5). The t‐SNE algorithm was run with the normalized expression matrix by RunTSNE function in Seurat package.

### In Vitro Co‐Culture Assay

Hepatic PD‐1^+^CD8^+^ or PD‐1^−^CD8^+^ T cells from dnTGFβRII Aire^−/−^ mice were isolated using a cell sorter and their cytotoxic potential evaluated. Briefly, 1 × 10^5^ of these CD8^+^ T cells were co‐cultured with 1 × 10^4^ primary hepatocytes that were isolated from wild‐type mice by perfusion or co‐cultured with 1 × 10^4^ AML12 cells in individual wells of a 96‐well plate, in the presence of 2 µg/ml anti‐CD3 (catalog 100 223, BioLegend) and 1 µg mL^−1^ anti‐CD28 antibody (catalog 102 116, BioLegend). Cells were cultured in RPMI 1640 medium (catalog C11875500BT, Gibco), supplemented with 10% FBS (catalog FSP500, Excell Bio), 1 mM Sodium Pyruvate (catalog 11 360 070, Gibco), 5 mM HEPES (catalog 15 630 080), 55 µM β‐mercaptoethanol (catalog PB180633, Procell) and 100 U mL^−1^ Penicillin‐Streptomycin (catalog 15 140 122, Gibco). The activity and morphology of cells were observed by high content imaging system (PerkinElmer Operetta CLS, Boston, MA) at 37 °C under 5% CO_2_. The LDH assay was carried out to detect the levels of LDH in the supernatant fluids after 16–24 h of co‐culture, using a LDH Cytotoxicity assay kit (catalog G1780, Promega), according to the manufacturer's instructions. To explore the function of perforin‐1, granzyme B and IFN‐γ on the induction of target cell pyroptosis, AML12 cells were treated with 10 µM nigericin (Invivogen) or a sublytic concentration of recombinant mouse perforin‐1 (Cloud‐Clone) and 500 nM granzyme B (R&D), or pretreated with 100 ng mL^−1^ IFN‐γ (Peprotech) for 24 h then treated with perforin‐1 and granzyme B with imaging at 18 h. In addition, 1 × 10^5^ PD‐1^+^CD8^+^ T cells were co‐cultured with 1 × 10^4^ AML12 cells treated with/without 50 µg mL^−1^ anti‐IFN‐γR antibody (BioXcell) or pretreated the PD‐1^+^CD8^+^ T cells with 100 µM DCI (Sigma‐Aldrich) for 1 h before co‐cultured with AML12 cells. Cell morphology was observed using a high content imaging system or a confocal microscopy. The cell culture supernatants were used to quantitate levels of LDH at 24 h. All in vitro experiments were repeated at least 3 times.

### FAM‐FLICA Caspase‐1 and PI Dual Staining Assay

To assess the activation levels of caspase‐1 and cell death in target cells during co‐culture of PD‐1^+^CD8^+^ T cells with AML12 or primary hepatocytes, FLICA caspase‐1 and PI dual staining were performed. The procedure followed the FAM‐FLICA caspase‐1 assay kit (ICT‐97, ImmunoChemistry Technologies) instructions. Briefly, after 24 h of co‐culture, the culture supernatant was removed, and 100 µL of 1 × FLICA working solution was added to each well of a 96‐well plate. The cells were incubated at 37 °C for 60 min, with gentle mixing every 20 min to distribute the reagent. After incubation, 1 µg mL^−1^ PI was added for 5 min, followed by three washes. Confocal fluorescence imaging or flow cytometry was performed after trypsinization to assess the results.

### Construction of CRISPR‐Cas9 Knockout AML12 Cells and In‐Vitro Co‐Culture Assay

To generate CRISPR‐Cas9 knockout AML12 cells, guide RNAs (gRNAs) targeting mouse GSDMD (#1: 5′‐AAAGTCTCTGATGTCGTCGA‐3′, #2: 5′‐CTGCAACAGCTTCGGAGTCG‐3′, #3: 5′‐TGCAACAGCTTCGGAGTCG‐3′) or a scrambled gRNA sequence (negative control: 5′‐GTGTAGTTCGACCATTCGTG‐3′), which does not target any sequence in both human and mouse genomes, were cloned into the gRNA expression plasmid pLV[CRISPR]‐hCas9/Puro‐U6. Lentivirus was then packaged and used to infect AML12 cells. 3 days after infection, puromycin (2 µg mL^−1^) was used to select positive cells harboring the pLV[CRISPR]‐hCas9/Puro‐U6 plasmid. Selected cells were then screened into single clones using the 96‐well plate limited dilution method. The single clones were cultured for an additional 21 days or longer, depending on the cell growth rate. The knockout effect was verified by gel electrophoresis experiments.

To explore the cytotoxicity of hepatic CD8^+^ T cells from dnTGFβRII Aire^−/−^ mice against GSDMD knockout or control AML12 cells, hepatic CD8^+^ T cells were enriched using CD8 (TIL) MicroBeads (Miltenyi Biotec) via magnetic‐activated cell sorting (MACS). Subsequently, 1 × 10^5^ of these CD8^+^ T cells were co‐cultured with 1 × 10^4^ GSDMD knockout or control AML12 cells in individual wells of a 96‐well plate, in the presence of 2 µg mL^−1^ pre‐coated anti‐CD3 (catalog 100 223, BioLegend) and 1 µg mL^−1^ soluble anti‐CD28 antibody (catalog 102 116, BioLegend). Cell morphology was imaged using a microscope, and the cell culture supernatants were used to quantitate levels of LDH after 24 h.

### Transwell Assay

A transwell assay was performed to quantitate the interaction of PD‐1^+^CD8^+^ T cells and AML12 cells. 1 × 10^6^ PD‐1^+^CD8^+^ T cells were added to an upper layer 24‐well insert with 0.4 µm membrane pore (catalog 3470, Corning), and 5 × 10^5^ AML12 cells were added to the bottom, after 72h, the cell morphology of AML12 in the center of bottom layer were imaging. Control group upper chambers were supplemented with hepatic CD8^+^ T cells from control mice for comparison.

### Adoptive Transfer Experiment of PD‐1^+^CD8^+^ T Cells

Hepatic PD‐1^+^ and PD‐1^−^CD8^+^ T cells were sorted from dnTGFβRII Aire^−/−^ mice (CD45.2 background) using a FACS Aria II cell sorter, 5 × 10^5^ PD‐1^+^ or PD‐1‐CD8^+^ T cells were adoptively transferred into CD45.1 Rag1^−/−^ mice by tail intravenous injection. After 12 weeks, recipient mice were sacrificed for detection of the percentage of PD‐1^+^CD8^+^ and PD‐1^−^CD8^+^ T cells in blood, liver, spleen, thymus, and salivary gland using flow cytometry.

### Manufacturing of CAR‐T Cells and PD‐1^+^ Cells Depletion Assay

Murine CD8^+^ T cells from WT mice or Pdcd1^−/−^ mice were enriched from single‐cell suspensions of dissociated spleens using CD8 (TIL) MicroBeads (Miltenyi Biotec) via magnetic‐activated cell sorting (MACS). Purified CD8^+^ T cells were activated with plate‐bound anti‐CD3 (4 µg mL^−1^) and anti‐CD28 antibodies (2 µg mL^−1^) in RPMI 1640 medium containing 10% fetal calf serum, sodium pyruvate (1 mM), penicillin–streptomycin (100 U mL^−1^), β‐mercaptoethanol (0.1 mM) and IL‐2 (100 U mL^−1^) for 2 days in 12‐well plates. On Day 3, the viral supernatant was transferred to RetroNectin‐coated 12‐well plates (TaKaRa). Activated CD8^+^ T cells were transduced by spin infection at 800 x g for 90 min. One day after transduction, the virus‐containing medium was replaced with fresh medium supplemented with 100 U mL^−1^ IL2, and the cells were cultured for two more days. Then, Thy1.1^+^ cells were positively selected with CD90.1 MicroBeads (Miltenyi Biotec) by MACS. Purified transduced T cells were further supplemented with IL‐7 and IL‐15 without IL‐2. On Day 7, cells were harvested for follow‐up experiments. Control T cells were processed with the same methods but transduced with empty vector. To deplete the PD‐1^+^ cells, 1‐week‐old dnTGFβRII Aire^−/−^ mice were intravenously injected with 5 × 10^5^ anti‐PD‐1 CAR‐T cells and monitored the diseases.

### In Vivo Administration of GSDMD Inhibitor

To investigate the role of the GSDMD‐mediated pyroptosis on disease progression, dnTGFβRII Aire^−/−^ mice were treated with disulfiram (DSF, Sigma‐Aldrich), which specifically inhibits pore‐formation of GSDMD.^[^ ^9^ ^]^ Briefly, 50 mg kg^−1^ of DSF was injected i.p. into dnTGFβRII Aire^−/−^ mice daily starting at 7 days of age. Survival was monitored and liver tissues collected for H&E staining.

### Protein Mass Spectrometry

To detect the differences of protein components in the culture supernatant of PD‐1^+^CD8^+^ T cells and control cells, protein mass spectrometry was utilized. Briefly, 3 × 10^5^ PD‐1^+^CD8^+^ T, PD‐1^−^CD8^+^ T cells were sorted from the livers of dnTGFβRII Aire^−/−^ mice or hepatic CD8^+^ T cells from dnTGFβRII Aire^+/−^ mice and cultured in vitro with serum‐free culture medium. After 24 h of culture, the culture supernatants were used for proteomic analysis. Proteins were extracted by adding lysis buffer and subjected to ultrasonication. After acidification and enzymatic digestion, protein peptides were obtained, dried in a vacuum centrifuge, and chromatographically separated using the nEASY LC1000 system (Thermo Fisher Scientific). The mobile phase consisted of 0.1% formic acid aqueous solution (A phase) and 0.1% formic acid acetonitrile aqueous solution (B phase). The samples separated by nanoscale liquid chromatography were collected using a timsTOF Pro2 mass spectrometer (Bruker) for data acquisition. The raw data from mass spectrometry analysis were identified using the Spetronaut database search software. Data filtering was performed with a standard of FDR < 0.01 to eliminate false positives, obtaining protein qualitative or quantitative data tables for subsequent analysis.

### Single Cell Sorting, Library Preparation, and Target Sequencing for Mouse Samples

To perform targeted single cell RNA sequencing of hepatic lymphocytes, BD Rhapsody Immune Response Panel Mm (BD Biosciences Kit Part Number: 633 753) was performed, which is a set of ready‐to‐use primer pools target to 397 immune‐related genes in mouse. Briefly, about 10 000 CD45^+^ lymphocytes were sorted from dnTGFβRII, Aire^−/−^, dnTGFβRII Aire^+/−^, and dnTGFβRII Aire^−/−^ mouse, respectively (each sample mixed with hepatic cells from 2–3 mice) using the FACS Aria II cell sorter. The cells of the four samples from different mice were then labeled with different sample tag (633 793, BD Single Cell Multiplexing Kit, BD Biosciences), which is an Anti‐Mouse CD45, Clone 30‐F11 antibody conjugated with a unique oligonucleotide sequence to allow for sample identification. Before loading cells to BD Rhapsody Cartridge, the cells from 4 samples were pooled together as one sample, and split to construct the TCR library, targeted mRNA library and sample tag library. Single‐cell library preparation was carried out according to the BD Rhapsody Single‐Cell Analysis System protocol (https://bdbiosciences.com/). About 20 000 cells from the cell suspension mixture were loaded onto a microwell array, followed by adding barcoded magnetic BD Rhapsody Cell Capture Beads and cell lysis buffer. Upon cell lysis, the mRNAs and sample tag barcodes of each cell were captured by probes via polyA/polyT hybridization. Beads were subsequently retrieved from the microwells by magnets and pooled into a single tube for reverse transcription to synthesize complementary DNAs (cDNAs). Then, cDNAs were amplified following multiple amplification schemes (https://bdbiosciences.com/). Amplified cDNAs were purified using SPRIselect beads (B23318, Beckman Coulter Life Sciences). Libraries were sequenced on NovaSeq 6000 (Illumina) after quality examination by Agilent 2100 Bioanalyzer (G2940CA, Agilent Technologies).

### Single Cell Targeted RNA‐Seq and TCR‐Seq Data Analysis

FASTQ files obtained from sequencing were provided to the BD Targeted Multiplex Rhapsody Analysis Pipeline (Version 1.9‐beta) with default settings. Putative cells information was combined together with DBEC‐adjusted molecules to generate a single‐cell expression matrix. The scRNA‐seq matrix with 14 202 cells × 397 genes was loaded in R language (Version 3.5.3) and converted to Seurat object using the CreateSeuratObject function in Seurat R package with default arguments (Version 3.1.5). Based on the number of sample tag reads obtained from the aligned results, 1084 “Multiplet” cells were filtered out that could detect two or more valid sample tags simultaneously, as well as 195 “Undetermined” cells that did not detect any sample tags (with reads for all sample tags less than 10). Then, genes were excluded that were expressed in less than 3 cells. This yielded a matrix with 385 genes and 12 923 cells, including 3462 cells from dnTGFβRII mice, 3480 cells from Aire^−/−^ mice, 3263 cells from dnTGFβRII Aire^+/−^ mice, and 2718 cells from dnTGFβRII Aire^−/−^ mice. This matrix was further analyzed using R and Seurat package (Version 3.1.5). First, the Seurat object was normalized and scaled by using the NormalizeData and ScaleData functions from the Seurat package (with default arguments). Next, RunPCA, FindNeighbors, FindClusters, and RunTSNE functions were sequentially run to perform dimensionality reduction and clustering. For the FindNeighbors and RunTSNE functions, the dims parameter was set to 1:30, and for the FindClusters function, the resolution parameter was set to 0.8. Ultimately, 21 distinct cell subpopulations were identified. Then, the subpopulations were defined based on the clustering results from the t‐SNE dimensionality reduction plots and the expression distribution of specific marker genes. Next, the T cells were filtered that have high expression of the *Cd3d* gene and do not express the *Klra1* gene. Finally, 2050 *Cd3d* ^+^ *Klra1* ^−^ T cells were used for further downstream analysis. After performing the same normalization, scaling, dimensionality reduction, and clustering processes on the T cell Seurat object as did with the whole cell data, 12 distinct T cell subpopulations were identified. For the T cell data, the dims parameter was set to 1:30 for both the FindNeighbors and RunTSNE functions, while the resolution parameter for the FindClusters function was set to 1.2. These subpopulations were further characterized based on the differential expression of marker genes, conducting gene expression feature analysis and functional assessments for each identified subsets. To integrate analysis of the scTCR‐seq data with the scRNA‐seq data, the TCR matrix of every single cell was imported in R, and was implemented into the metadata slot of a seurat object of T cells and used for further analysis.

Gene set variation analysis (GSVA) was performed using the GSVA package (Version 1.36.2). Gene sets were downloaded from MSigDB.^[^ ^38^ ^]^ The manually designed gene sets included “Immune checkpoints” gene set which contains 21 genes, *Lag3, Pdcd1, Tigit, Ctla4, Havcr2, Cd244, Tox, Cd74, Tnfrsf4, Tnfrsf9, Id2, Il10, Lgals3bp, Vhl, Prdm1, Btla, Hmgb3, Gnrh1, Adora2a, Anxa1, Cd44*, and “Pyroptosis” which contains 20 genes, *Gsdme, Gsdmd, Casp1, Casp3, Il1b, Il18, Aim2, Apip, Casp4, Naip2, Naip1, Naip5, Naip6, Naip7, Nlrc4, Nlrp1b, Mefv, Pycard, Il18r1, and Nlrp3*. The difference in pathway enrichment score per cell between different groups was calculated using the LIMMA package (Version 3.44.3).

### Statistical Analysis

Statistical significance was analyzed using GraphPad Prism 8 (GraphPad Software, San Diego, CA). Results in all figures are expressed as mean ± SD. For comparison of data from multiple groups or two groups of mice, statistics were analyzed using one‐way ANOVA or unpaired two‐tailed student's *t* test respectively. For survival study, log‐rank test was used to determine the statistical significance. Non‐parameteric Wilcoxon rank sum test was performed to assess the statistical significance in single‐cell RNA sequencing data. The statistical significance is reflected as *p < 0.05; **p < 0.01; ***p < 0.001.

## Conflict of Interest

The authors declare no conflict of interest.

## Author Contributions

J.L. and S.Y.Y. contributed equally to this work. Z.X.L. and Z.B.Z. conceived and supervised the project. M.E.G. and Y.H. supported the conceptualization and supervision. J.L. and S.Y.Y. designed and performed the majority of experiments. Z.H.B., H.X.Z., M.M., X.Q.W., and L.L. helped with the biological experiments and mouse experiments. W.Z. helped with the methodology and offered technical support. J.L. and S.Y.Y. analyzed the data and wrote the manuscript. All authors reviewed and edited the manuscript

## Supporting information



Supporting Information

Supplemental Movie 1

## Data Availability

The bulk RNA‐seq data and scRNA‐seq datasets generated in this study are available for download at the Gene Expression Omnibus (GEO): GSE199065. Any additional information required to reanalyze the data reported in this paper is available from the lead contact upon reasonable request.
