# Supplementary material for: PD‐1+CD8+ T Cell‐Mediated Hepatocyte Pyroptosis Promotes Progression of Murine Autoimmune Liver Disease
Source: Adv Sci (Weinh). 2024 Nov 4;12(1):2407284. doi: 10.1002/advs.202407284 (PMC11714232; doi:10.1002/advs.202407284)
Supplement: Supplementary file 1 — Supporting Information [file ADVS-12-2407284-s002.pdf]

ADVANCED SCIENCE
Open Access

# Supporting Information

for *Adv. Sci.*, DOI 10.1002/advs.202407284

PD-1$^+$CD8$^+$ T Cell-Mediated Hepatocyte Pyroptosis Promotes Progression of Murine Autoimmune Liver Disease

*Jie Long, Si-Yu Yang, Zhen-Hua Bian, Hao-Xian Zhu, Min Ma, Xiao-Qing Wang, Liang Li, Weici Zhang, Ying Han*, M. Eric Gershwin*, Zhe-Xiong Lian* and Zhi-Bin Zhao**

Supporting Information

**PD-1$^+$CD8$^+$ T Cell-Mediated Hepatocyte Pyroptosis Promotes Progression of Murine Autoimmune Liver Disease**

*Jie Long, Si-Yu Yang, Zhen-Hua Bian, Hao-Xian Zhu, Min Ma, Xiao-Qing Wang, Liang Li, Weici Zhang, Ying Han,* M. Eric Gershwin,* Zhe-Xiong Lian,* Zhi-Bin Zhao**

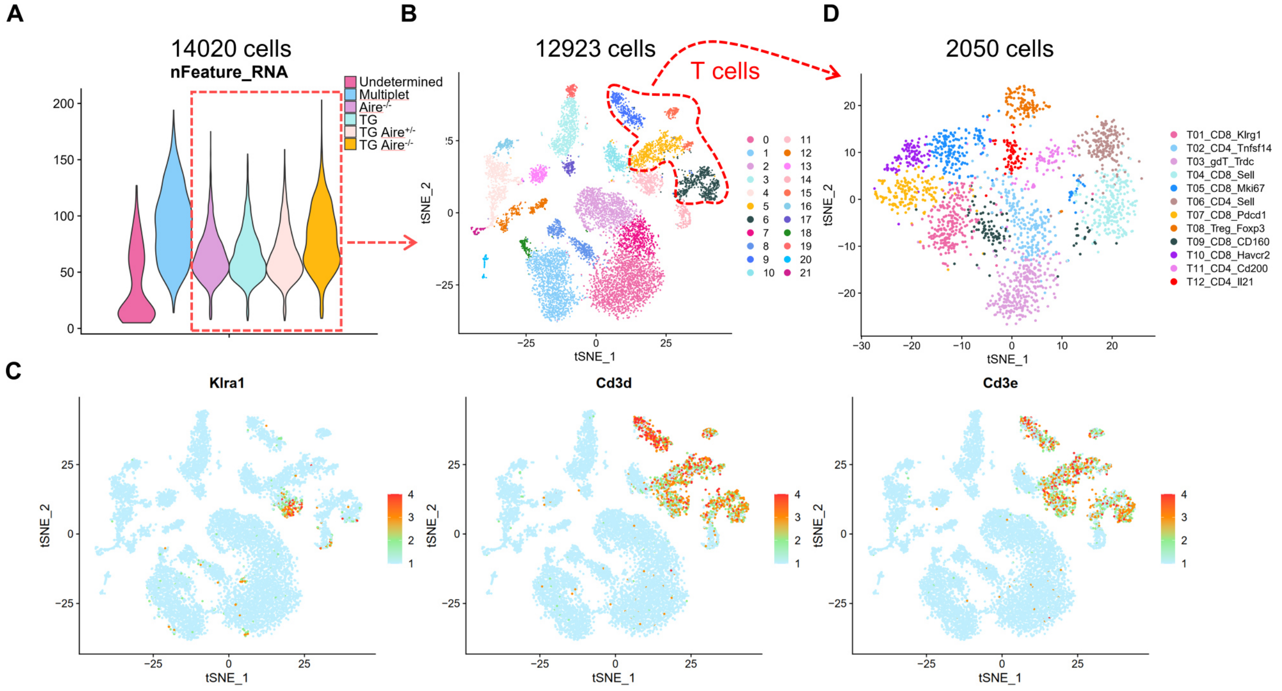


**Figure S1. The quality control and filtering processes for single-cell sequencing data**. (**A**) Violin plot displays the distributions of the numbers of detected genes (i.e., nFeature_RNA) in four single-cell RNA sequencing samples from the livers of Aire$^{-/-}$, TG, TG Aire$^{+/-}$, and TG Aire$^{-/-}$ mice, as well as in the cells inferred as "Undetermined" and "Multiplet" through sample tags. (**B**) t-SNE plots of 12923 CD45$^{+}$ cells from 4 liver samples of Aire$^{-/-}$, TG, TG Aire$^{+/-}$ and TG Aire$^{-/-}$ mice. (**C**) Expression distributions of *Klra1*, *Cd3d* and *Cd3e* on t-SNE plots of CD45$^{+}$ cells. (**D**) t-SNE plots of 2050 *Cd3d*$^{+}$*Klra1*$^{-}$ T cells selected from (B).

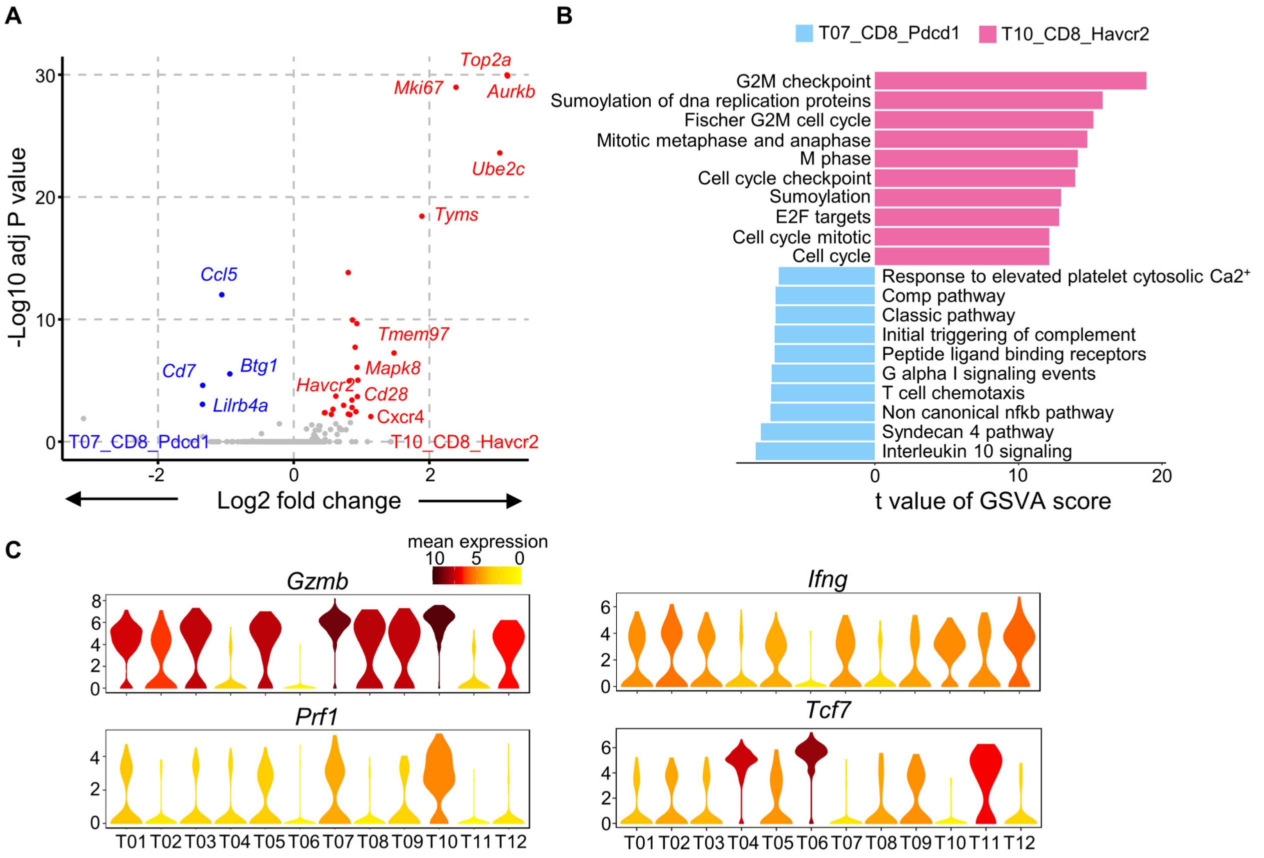


**Figure S2. Function analysis of different Pdcd1⁺CD8⁺ T cell clusters**. (**A**) Volcano plot compared the differential expressed genes between T07_CD8_Pdcd1 and T10_CD8_Havcr2 clusters. (**B**) GSVA analysis displayed the top 20 different enriched pathways between T07_CD8_Pdcd1 and T10_CD8_Havcr2 clusters. (**C**) Violin plots showed the expression levels of *Prf1*, *Gzmb*, *Ifng* and *Tcf7* in 12 different clusters of T cells. A non-parameteric Wilcoxon rank sum test was performed to assess the statistical significance in (A).

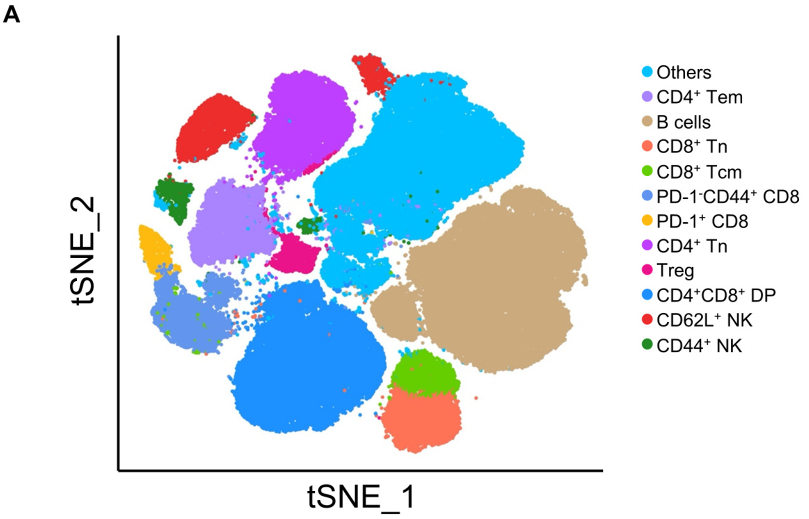


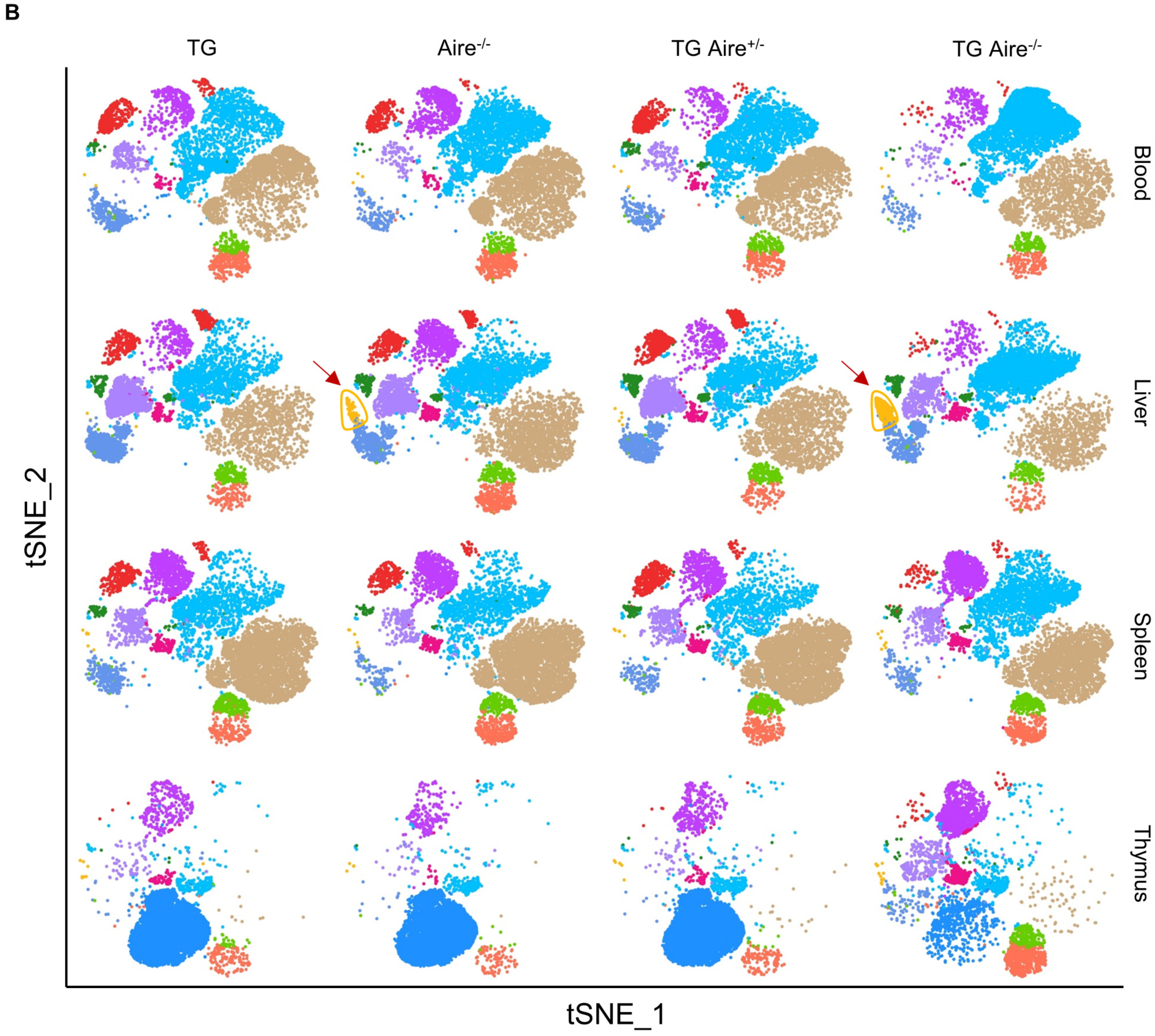


**Figure S3. t-SNE plots of lymphocytes in multiple organs.** (**A**) Merge and (**B**) split t-SNE plots of $CD45^+$ immune cells in the blood, liver, spleen and thymus from TG, $Aire^{-/-}$, TG $Aire^{+/-}$ and TG $Aire^{-/-}$ mice.

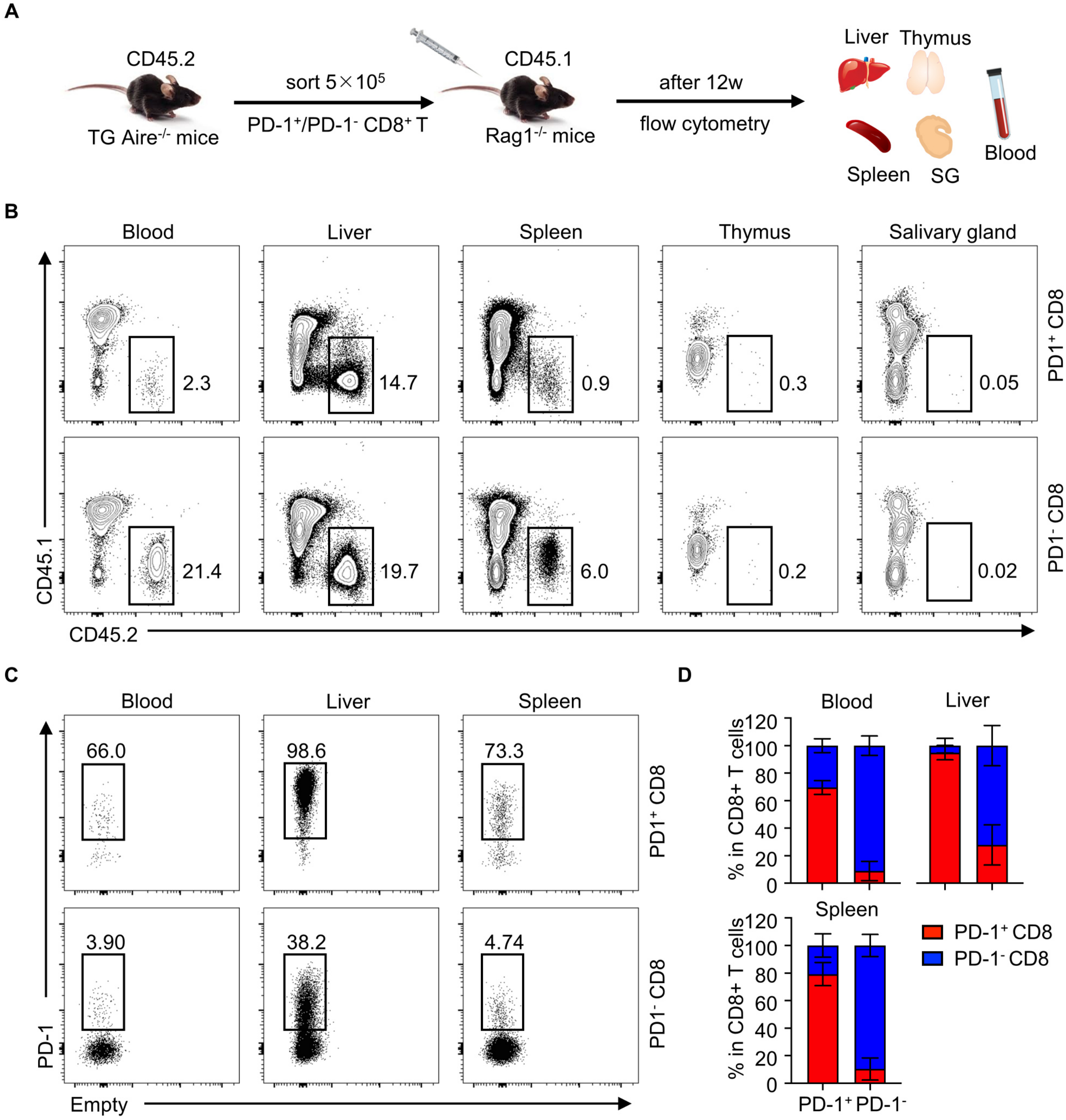


**Figure S4. Transferred PD-1⁺CD8⁺ T cells specifically home to the liver.** (**A**) Schematic diagram of the CD8⁺ T cells adoptive transfer experiment. 5×10⁵ PD-1⁺ or PD-1⁻CD8⁺ T cells were sorted from the livers of TG Aire⁻/⁻ mice and transferred into CD45.1 Rag1⁻/⁻ mice. (**B**) Flow cytometry results show the percentage of CD45.2⁺ transferred PD-1⁺CD8⁺ and PD-1⁻CD8⁺ T cells in the blood, liver, spleen, thymus and salivary gland of recipient mice after 12 weeks. (**C**) Flow cytometry results show the percentage of PD-1⁺CD8⁺ T cells in the blood, liver and spleen on the gated population of CD45.2⁺CD8⁺ T cells. (**D**) Stacked barplots show the relative frequencies of PD-1⁺CD8⁺ and PD-1⁻CD8⁺ T cells in the blood, liver and spleen.

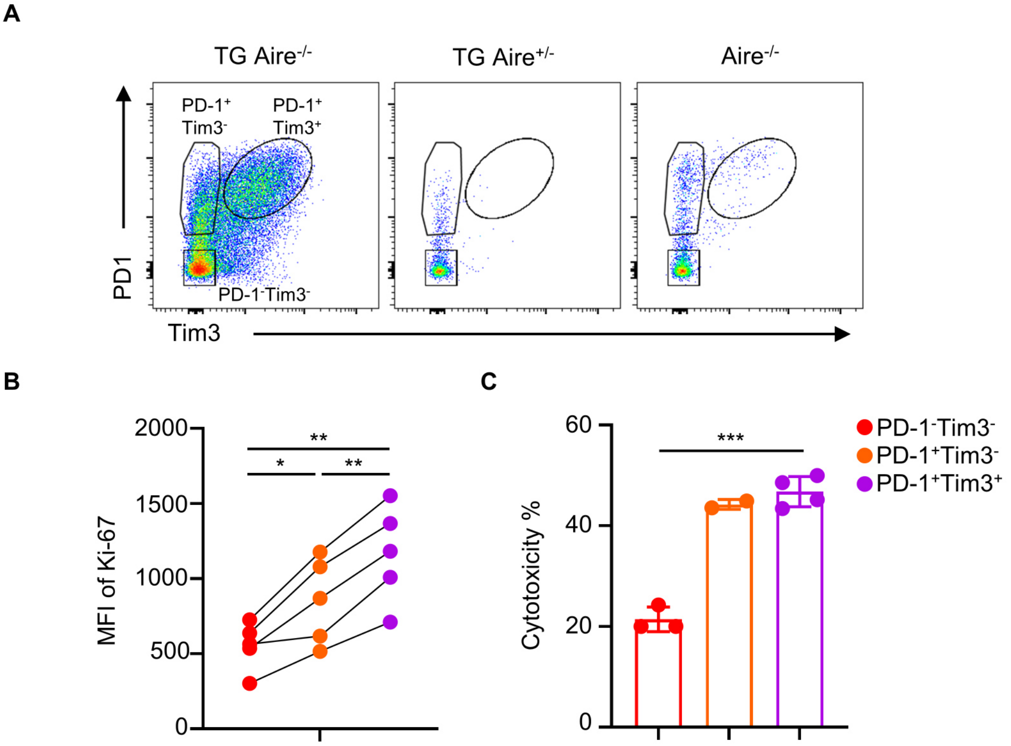


**Figure S5. Function detection of different PD-1$^+$CD8$^+$ T cell clusters**. (**A**) Flow cytometry results show the PD-1$^-$Tim3$^-$, PD-1$^+$Tim3$^-$ and PD-1$^+$Tim3$^+$ clusters in the livers of Aire$^{-/-}$, TG Aire$^{+/-}$ and TG Aire$^{-/-}$ mice on the gated population of CD45.2$^+$CD3$^+$NK1.1$^-$CD8$^+$ T cells. (**B**) Statistical analysis of the MFI of Ki-67 in PD-1$^-$Tim3$^-$, PD-1$^+$Tim3$^-$ and PD-1$^+$Tim3$^+$ CD8$^+$ T cells from the livers of TG Aire$^{-/-}$ (n=5) mice. (**C**) Statistical analysis of the cytotoxicity of 1×10$^5$ PD-1$^-$Tim3$^-$ (n=3), PD-1$^+$Tim3$^-$ (n=2) and PD-1$^+$Tim3$^+$ CD8$^+$ T cells (n=4) following co-culture with 1×10$^4$ isolated primary hepatocytes from wild-type mice in vitro, detected the levels of LDH in supernatants and calculated the cytotoxicity percentages after 20 hr. Data are means ± SD. *P < 0.05; **P < 0.01; ***P < 0.001, by matching or no matching one-way ANOVA (B and C).

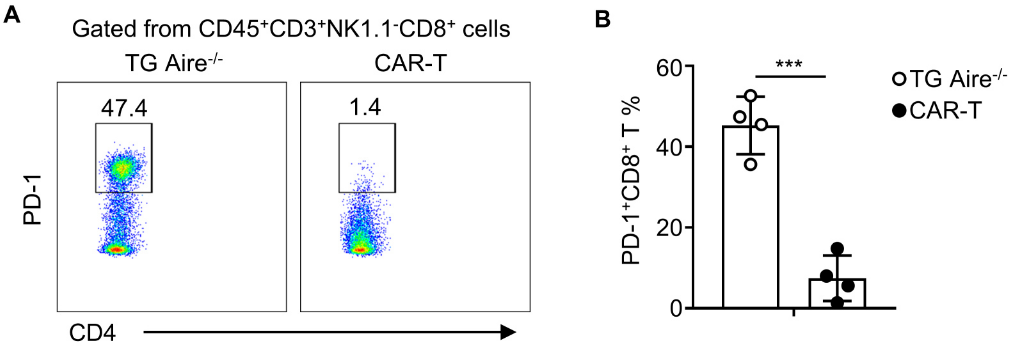


**Figure S6. Depletion of hepatic PD-1$^+$CD8$^+$ T cells with PD-1 targeting CAR-T cells.** (**A**) Flow cytometry results show the expression of PD-1 in the liver of TG Aire$^{-/-}$ mice with or without CAR-T treatment. (**B**) Statistical analysis of the percentage of PD-1$^+$CD8$^+$ T cells in the liver of TG Aire$^{-/-}$ mice with (n=4) or without (n=4) CAR-T treatment. Data are means ± SD. ***$P < 0.001$, by unpaired Student's t test.

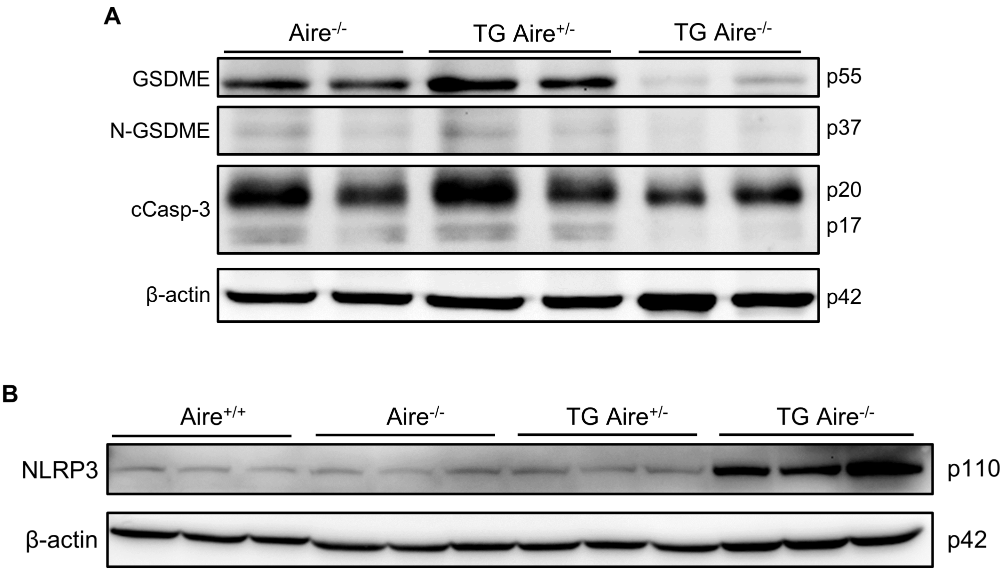


**Figure S7. Induced pyroptosis of hepatocyte of dnTGFβRII Aire$^{-/-}$ mice is independent on GSDME.** (**A**) Detection of the expression levels of GSDME and caspase-3 (cleaved) in the liver tissues of Aire$^{-/-}$, TG Aire$^{+/-}$ and TG Aire$^{-/-}$ mice by Western blot using anti-GSDME antibody (ab215191) and anti-Caspase-3 antibody (sc-56053). β-actin is the same band in Figure 6A as a single control in one experiment. (**B**) Detection of the expression levels of NLRP3 in the liver tissues of Aire$^{+/+}$, Aire$^{-/-}$, TG Aire$^{+/-}$ and TG Aire$^{-/-}$ mice by Western blot using anti-NLRP3 antibody (AG-20B-0014-C100) .

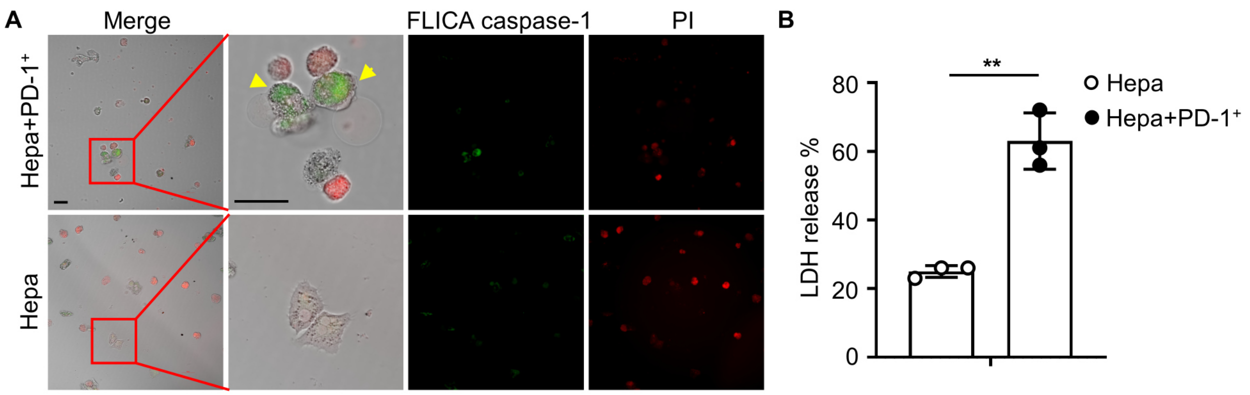


**Figure S8. PD-1$^+$CD8$^+$ T cellls induce pyroptosis of primary hepatocyte.** (**A**)$1\times10^4$ isolated primary hepatocytes from wild type mice were co-cultured with/without $1\times10^5$ PD-1$^+$CD8$^+$ T cells, and labeled with FAM-FLICA caspase-1 and PI after 24h, then the cell morphology and fluorescence signal were detected by a fluorescence confocal microscopy. Yellow arrows show hepatocytes in the process of pyroptosis. Scale bars, 50 μm. (**B**) The culture supernatants were collected after 24 hours for LDH detection and calculation of the release rate. Data are means ± SD. **P < 0.01, by unpaired Student's t test.

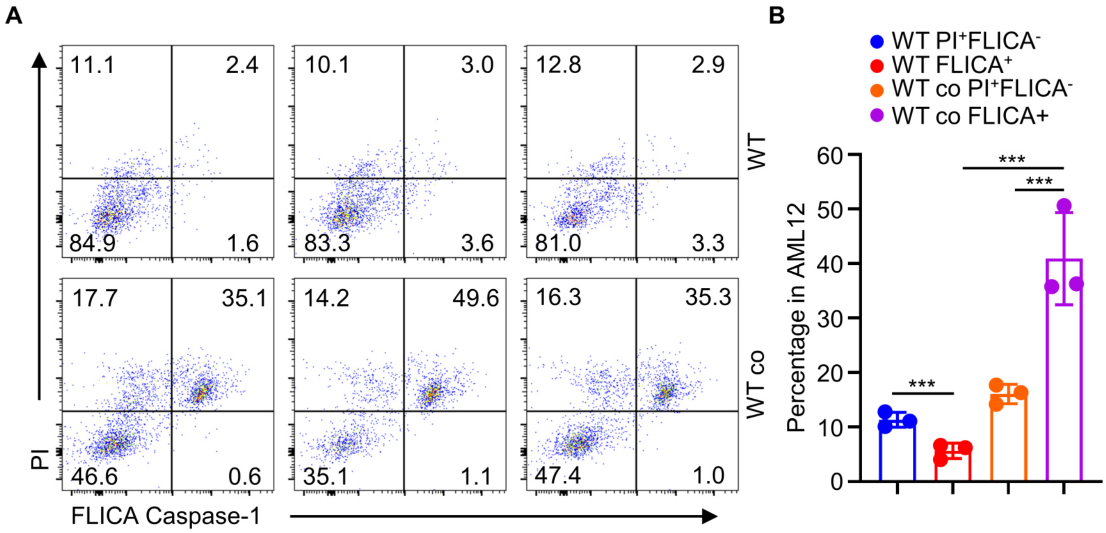


**Figure S9. PD-1⁺CD8⁺ T cells induce the activation of caspase-1 in target cells.** (**A**)$1\times10^4$ wild type AML12 were co-cultured with/without $1\times10^5$ PD-1$^+$CD8$^+$ T cells, and labeled with FAM-FLICA caspase-1 and PI after 24h, then the cells were digested and detected the fluorescence signal using flow cytometry. Flow cytometry results show the fluorescence signal intensity of FLICA caspase-1 and PI. (**B**) Statistical analysis of the percentage of FLICA caspase-1$^+$ and PI$^+$FLICA caspase-1$^-$ AML12 in total AML12 cells. WT co refers to wild type AML12 cells co-cultured with PD-1$^+$CD8$^+$ T cells. Data are means $\pm$ SD. ***$P < 0.001$, by one-way ANOVA.

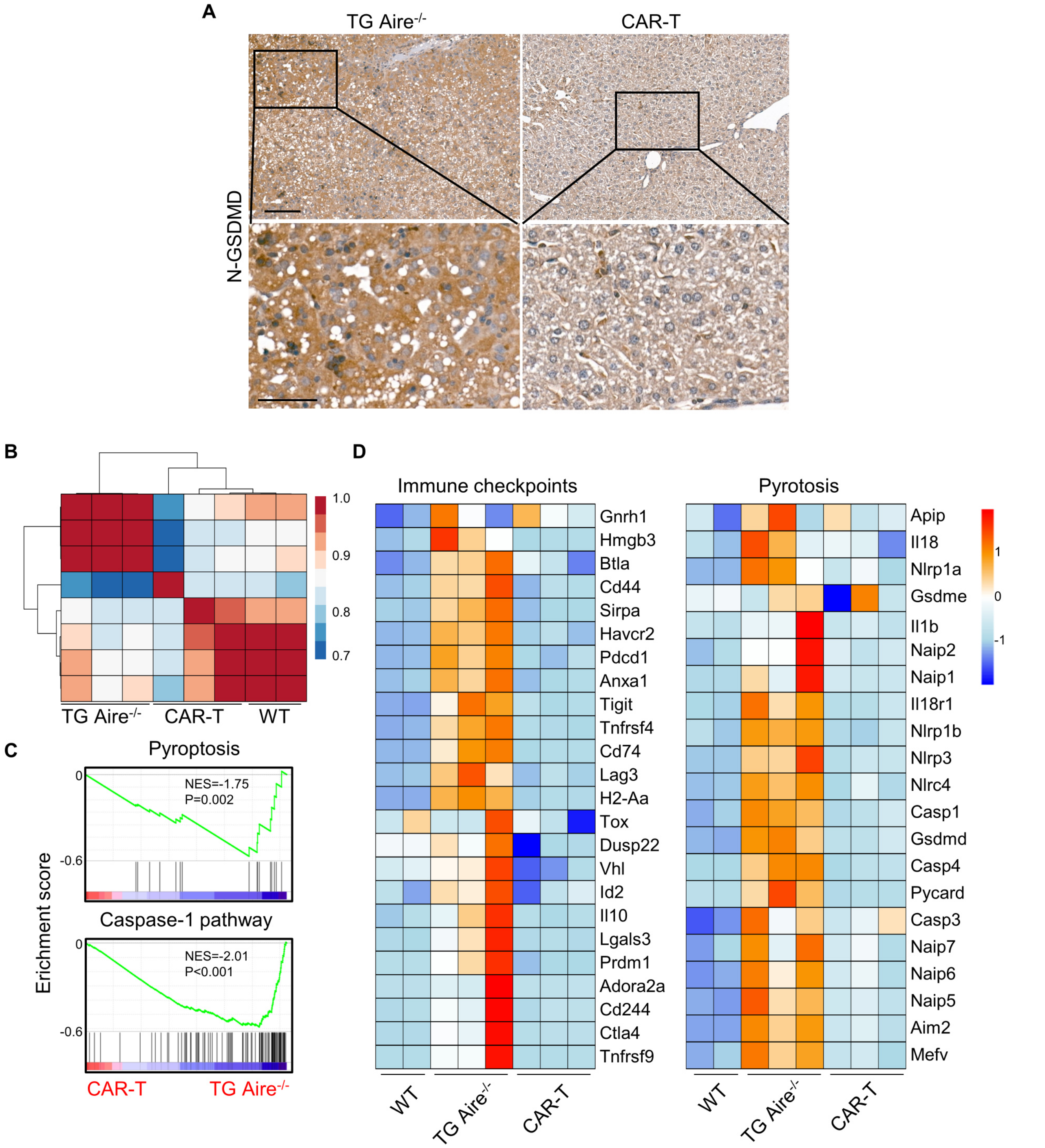


**Figure S10. PD-1 targeting CAR-T cells inhibit the pyroptosis of hepatocytes.** (**A**) Immunohistochemistry results of the expressions of N-GSDMD in the liver tissues from PD-1 targeting CAR-T cells treated or untreated TG Aire$^{-/-}$ mice. Scale bar in the upper row, 100 μm; scale bar in the bottom row, 50 μm. (**B**) The Pearson correlation coefficient heatmap displays the correlations between RNA-seq samples from the livers of WT and TG Aire$^{-/-}$ mice, as well as CAR-T treated TG Aire$^{-/-}$ mice. (**C**) GSEA analysis shows the enrichment of pyroptosis and caspase-1 pathways in TG Aire$^{-/-}$ mice with or without CAR-T treated. (**D**) Heatmap results show expressions of immune checkpoints and pyroptosis pathway-related genes in liver tissues from WT, TG Aire$^{-/-}$ mice, and CAR-T treated TG Aire$^{-/-}$ mice.

**Table S1. The detailed information of top 10 TCR clonotypes in CD8$^+$ T cells**

| TCR clonotype | TCR CDR3 (aa sequence) | TCRα sequence | TCRβ sequence | Cell number | Frequency |
|---|---|---|---|---|---|
| Clonotype1 | AMNNNAGAKLT\|\|ASSTGGSDYT | AMNNNAGAKLT | ASSTGGSDYT | 59 | 0.0363 |
| Clonotype2 | AMNNNAGAKLT\|\|ASSLGGSDYT | AMNNNAGAKLT | ASSLGGSDYT | 21 | 0.0129 |
| Clonotype3 | AAEHYGSSGNKLI\|\|ASSTGGEQY | AAEHYGSSGNKLI | ASSTGGEQY | 12 | 0.0074 |
| Clonotype4 | AAENYGSSGNKLI\|\|ASSTGGEQY | AAENYGSSGNKLI | ASSTGGEQY | 10 | 0.0062 |
| Clonotype5 | AASAQWRQQLQTD\|\|ASSTGGSDYT | AASAQWRQQLQTD | ASSTGGSDYT | 9 | 0.0055 |
| Clonotype6 | VLSASMGYKLT\|\|ASSHNAEQF | VLSASMGYKLT | ASSHNAEQF | 9 | 0.0055 |
| Clonotype7 | AAEDYGSSGNKLI\|\|ASSTGGEQY | AAEDYGSSGNKLI | ASSTGGEQY | 8 | 0.0049 |
| Clonotype8 | AMREGLGTGSKLS\|\|ASSSQNTLY | AMREGLGTGSKLS | ASSSQNTLY | 8 | 0.0049 |
| Clonotype9 | SYGYSSGFHKV\|\|ALMELGGIRATDKLV | SYGYSSGFHKV | ALMELGGIRATDKLV | 8 | 0.0049 |
| Clonotype10 | ACWDSSGFHKV\|\|GSDIGGSSWDTRQMF | ACWDSSGFHKV | GSDIGGSSWDTRQMF | 7 | 0.0043 |
| Others | Others | Others | Others | 1473 | 0.907 |
